# Barriers to managing clinical alarms in intensive care units: a cross-sectional study

**DOI:** 10.1590/1518-8345.7969.4843

**Published:** 2026-06-15

**Authors:** Renata Cristina Crippa Diekmann, Lillian Caroline Fernandes, Ramon Antonio Oliveira

**Affiliations:** 1 Sociedade Israelita Brasileira Albert Einstein, Faculdade Israelita de Ciências da Saúde Albert Einstein, São Paulo, SP, Brazil.; 2 Universidade de São Paulo, Escola de Enfermagem, São Paulo, SP, Brazil.

**Keywords:** Intensive Care Units, Critical Care, Patient Safety, Clinical Alarms, Nurse Practitioners, Nursing

## Abstract

**Objective:**

to investigate the barriers to clinical alarm management according to nurses and nursing technicians and to examine characteristics of clinical alarm response in intensive care units.

**Method:**

a cross-sectional study was conducted in a hospital accredited by the Joint Commission International and the American Nurses Credentialing Center Magnet Recognition. A questionnaire was used to identify barriers to clinical alarm management and 84 hours of observation were carried out.

**Results:**

92 professionals participated (33 nurses; 59 nursing technicians). The perception of inadequate nursing staffing levels was more frequent among nurses (21.2%) than among nursing technicians (3.4%; p=0.006). Competition between clinical alarms and noise from other equipment was identified as similar among nurses (20; 60.6%) and nursing technicians (35; 53.9%; p=0.981). Frequent interruptions of activities were reported by nurses (6; 18.2%) and nursing technicians (17; 28.8%) (p=0.615). Forty-nine clinical alarms were recorded, with a mean response time of 2.5 minutes (SD 2.8) and three (6.1%) were ignored.

**Conclusion:**

several barriers to adequate management were identified, including human resources, environmental factors and work processes. Regarding care, alarms from multiparameter monitors predominated, responded to mainly by the nursing staff, with a low proportion of ignored clinical alarms.

## Highlights

**(1)** The study investigated barriers to the management of clinical alarms in the Intensive Care Units. **(2)** Staff shortages were cited as a barrier by 21.2% of nurses. **(3)** Excessive noise was considered a problem by 60.6% of nurses. **(4)** Excessive noise was considered a barrier by 59.3% of nursing technicians. **(5)** 6.1% of clinical alarms were ignored by nurses and nursing technicians.

## Introduction

Intensive care units (ICUs) are intended for the care, under continuous surveillance, of critically ill or at-risk patients who are potentially recoverable, through diagnosis, treatment and rehabilitation, provided by a multidisciplinary team composed of physicians, nurses, nursing technicians, physiotherapists, pharmacists, nutritionists and psychologists who promote person-centered care^1^ ^-^ ^3^.

To provide care in this setting, it is necessary to employ various health technologies, such as medical devices^4^. Among them, multiparameter monitors, mechanical ventilators, continuous infusion pumps and pulse oximeters stand out, for example. These devices collect data from the patient or from certain therapies administered to him at high frequency, that is, they read heart and respiratory rate, peripheral oxygen saturation, temperature and drug or medical gas administration conditions multiple times per second^5^. Medical devices are considered clinical decision support devices and, due to their relevance, are equipped with clinical alarm (CA) systems^6^.

CAs can be defined as visual and auditory resources present in various medical devices in order to maintain patient safety. They must be configured according to the clinical characteristics and needs of each patient, in order to avoid false-positive alarms, as well as adverse events (AEs) resulting from unsignaled situations^7^. In this sense, CAs are important tools that can contribute to improving patient safety standards, as certain AEs and incidents that result in harm to health can be avoided through the management of CAs emitted by medical devices^8^.

CA management comprises a combination of evidence-based interventions, including educational programs, policy and procedure development, reduction of excessive patient monitoring and personalization of CA parameters^9^. Underlining its importance, this topic was incorporated in 2019 into the 7th edition of the Joint Commission International (JCI) International Manual of Standards for Hospital Accreditation, described in the Patient Care chapter^10^.

Several aspects can harm CA management, such as the lack of uniformity in monitoring standards, the absolute prioritization of patients with greater severity to the detriment of those who require care less frequently or whose illnesses suggest less severity, the understaffing of nursing professionals and CA fatigue^11^. Results from national and international studies that have investigated CA management have focused mainly on elucidating the effects of alarm fatigue among nursing assistants in ICUs^11^ ^-^ ^13^. It has been shown that nurses are exposed to high levels of alarm fatigue, which causes a high frequency of ignored CAs and a relationship between alarm fatigue and burnout^11^ ^-^ ^13^.

It should be noted, however, that the category of nursing technicians or nursing assistants, which constitutes an important part of nursing care providers in ICUs, has not been included, which ends up limiting the analysis of CA management in national and international ICUs^11^ ^-^ ^13^. Thus, although the scientific literature points to deleterious effects of the absence of CA management, there is still a need for investigations that provide an understanding of the factors that affect and hinder the proper management of CA, based on the principles of patient safety, among members of the nursing team^14^.

Therefore, taking the statement into consideration, this study aimed to investigate the barriers to clinical alarm management according to nurses and nursing technicians and to examine characteristics of clinical alarm response in intensive care units. 

## Method

### Study design

This is a cross-sectional study reported according to the Strengthening the Reporting of Observational Studies in Epidemiology guidelines (STROBE)^15^.

### Study setting

The study was conducted in three ICUs of a large, private, philanthropic general hospital located in the city of São Paulo, SP, Brazil and accredited by the Joint Commission International and the American Nurses Credentialing Center Magnet Recognition, with 966 beds, including medical and surgical wards, a semi-intensive care unit, and adult, neonatal, and pediatric intensive care units. The 84 adult intensive care beds are distributed across three units, as follows: ICU A, general, serving patients with clinical or surgical conditions; ICU B, specializing in the care of patients with neurological conditions and ICU C, specializing in the care of liver transplant recipients.

### Population and sample

The population consisted of male and female nurses and nursing technicians working in the ICUs; in these units selected for the investigation, there were 230 nurses and nursing technicians. Thus, in order to guarantee the representativeness of the investigation results, a non-probabilistic convenience sample was established^16^. Of the 230 professionals accessible to the researchers, 30 were absent or on vacation. Therefore, the population consisted of 200 professionals. 

### Eligibility criteria

Nurses and nursing technicians of both sexes, aged 18 years or older, working in the ICUs of the institution selected for the study were included, regardless of their length of training or professional experience, from all shifts and who agreed to participate in the research by signing the Informed Consent Form (ICF). 

Professionals on vacation or leave during the data collection period were excluded.

### Study variables

#### Variables related to professionals

Socio-professional characterization variables, namely: sex, marital status, professional category, educational background (BA, postgraduation), area of expertise, work shift, presence of dual professional employment and post-shift work status.

#### Variables related to CA

Variables related to CA activation and response were collected, namely: medical device where the CA was activated, shift in which the alarm was triggered, parameter associated with the CA activation, time elapsed between CA activation and response (response time) and occurrence of ignored CAs. The CAs which were not responded to for a period equal to or greater than five minutes were considered ignored.

#### Variables and data collection instrument related to barriers to CA management

The data collection instrument was edited by the authors taking into account the variables used in a previous study^17^ and based on the recommendations of the Healthcare Technology Foundation^18^. Thus, nine variables were included, namely: occurrence of false-positive alarms that lead to reduced attention or response to CA; difficulties in detecting or understanding the priority of an CA; adequacy of nursing staff sizing for CA care; difficulty in hearing CAs when they are triggered; difficulty in identifying the origin of an CA; excessive dependence on CAs to draw attention to patient problems; competition between CA noises and non-clinical equipment; lack of training in CA systems; difficulty in configuring CAs correctly. For each of these, nursing professionals were asked to answer whether or not they observed its occurrence.

### CA management procedures in the ICUs selected for the study

In ICU A, the beds are arranged in private rooms and equipped with individual monitors. The supervision of the multiparameter monitors is carried out at two points: at the nursing station and in the pivot room. The nursing station has simultaneous mirroring of the multiparameter monitor screens on a video monitor and the pivot room, a specific physical space located within the ICU, is equipped with two large video monitors. The first simultaneously mirrors the screens of each patient’s multiparameter monitors and the second displays images captured from each room, allowing identification of when a professional attends to an CA, for example. These monitors are constantly observed by a nurse or nursing technician. The professionals are trained to recognize changes in vital signs, as well as in electrocardiographic tracings. When they identify changes, they notify the nursing team members responsible for direct patient care by telephone.

In ICUs B and C, the beds are separated by curtains or cubicles. In these settings, multiparameter monitors and other devices are directly observed by the nursing staff, without supervision of the signals captured by the multiparameter monitors by a nursing professional in a dedicated room. In all ICUs, the minimum and maximum limits configured on the ACI (Acute Coronary Intervention) devices are determined and adjusted daily, according to the clinical characteristics of each patient, by the on-call nurses and physicians. Thus, according to institutional policy, the nurse is the professional responsible for adjusting the ACI parameters on the multiparameter monitors and continuous infusion pumps in the ICUs.

### Data collection

#### Data collection from CAs

The data collection phase for CAs from multiparameter monitors, continuous infusion pumps and mechanical ventilators took place from June 7 to August 16, 2024 and the data collection phase for nursing professionals took place from June 27 to September 20, 2024.

The following steps were followed: the ICU beds were selected randomly, that is, the bed numbers were drawn using an alphanumeric list previously established by the researchers and placed in an opaque envelope. After drawing four beds, the researchers recorded the response time of the CAs by the professionals on a data collection form using a stopwatch. To measure the response time to the CA, it was decided to perform direct observation of one medical device per step, since it would not be possible to perform this procedure considering several medical devices simultaneously. For this purpose, 84 hours of observation were carried out in order to estimate the frequency of CA activation according to each medical device, namely: multiparameter monitor, continuous infusion pump and mechanical ventilator.

To record the reasons for CA activation, bed number and response time, the following procedures were adopted:

- In ICU A, the researchers, one per shift, remained in the pivot room and monitored the care provided to the patients using the multiparameter monitors via video monitors that displayed both the mirror image of each device and the images obtained through the cameras positioned in the patient rooms.
- In ICUs B and C, after the beds were randomly assigned, the researchers remained, on all shifts, positioned at the nursing stations, one per shift, which facilitated the visualization of the devices and the care provided to the patients.

No CA activations resulting from disconnecting one of the cables for patient repositioning in bed, bed baths, interruption of monitoring during patient movement to the toilet, ambulation, or electrode replacement were recorded. The respiratory rate parameter was not considered in the data collection process due to its standard procedure of being silenced in the units selected for the study.

#### Data collection from nursing professionals

All professionals were invited individually by the researchers, according to their availability at the nursing stations of each ICU. To ensure broad coverage, the approach occurred during all work shifts (morning, afternoon, and night). At these times, the researchers presented the objectives of the study and formally invited them to participate. This procedure was maintained until all professionals were included.

The professionals were instructed not to answer the form together or discuss their answers with colleagues. Anonymity was guaranteed at all stages of the study. Subsequently, each participant individually received an anonymized form containing questions about socio-professional characteristics and barriers to CA management. Collection took place at a later date, according to each professional’s preference.

### Storage, processing and analysis of data

After data collection on anonymized physical forms, the data were transcribed and stored in a way that ensured the secrecy, confidentiality, and privacy of the research participants using RedCap^®^ software (Nashville, TN, USA). Subsequently, the data were exported to RStudio^®^ software version 1.2.5019 (RStudio, Boston, MA, USA) and kept in a password-protected spreadsheet, under the researchers’ custody, on an institutional microcomputer.

The measures of central tendency, mean and standard deviation or median and interquartile range of the numerical variables were computed, and then the absolute and relative frequencies of the categorical variables were calculated, with their respective 95% confidence intervals. To the distribution of the numerical variables, the Shapiro-Wilk test and inspection of the Q-Q (Quantile-Quantile) plots were used. It was found that the numerical variables have a distribution different from the normal distribution, which is why the non-parametric Wilcoxon-Mann-Whitney test was used for comparison between groups. The existence of an association between two categorical variables was verified using Pearson’s Chi-square test or, when the contingency tables presented expected values of less than five cases, Fisher’s Exact test.

For all hypothesis tests, a significance level of α = 5% was used.

### Ethical aspects

The study was approved by the Research Ethics Committee of the hospital selected to conduct the study, under Protocol No. 6.800.354. The Guidelines and Regulatory Standards for Research involving human beings, issued by Brazilian National Health Council Resolution No. 466/12^19^, were respected. 

## Results

Two hundred nursing professionals were invited to participate in the study. Of these, 92 (46.0%) participated in the study. Regarding professional category, 33 (35.9%) were nurses and 59 (64.1%) were nursing technicians.

The majority of professionals were female (60; 65.2%), with a median age of 34 (33.5-37.1) years. Most self-identified as married (46; 50.0%) and with children (49; 53.3%). The median length of professional experience in the ICU was 5 (6.0-8.1) years. Among the nurses, 28 were ICU specialists (84.8%) and one professional had a master’s degree in nursing (3.0%). Regarding work shifts, it was found that 45 (48.9%) of the professionals worked in the morning, 43 (46.8%) in the afternoon, and 39 (42.4%) in the night (Table 1).

**Table 1:** Sociodemographic and professional characteristics of nurses and nursing technicians working in general and specialized intensive care units (n* = 92). São Paulo, SP, Brazil, 2024

Variables	n*	%
	(n*= 92)	
Sex		
Male	30	32.6
Female	60	65.2
Not informed	2	2.2
Marital status		
Single	37	40.2
Married	46	50.0
Divorced	8	8.7
Not informed	1	1.1
With children	49	53.3
Professional category		
Nurse technician	59	64.1
Nurse	33	35.9
Shift they answered		
Morning	15	16.3
Afternoon	40	43.5
Evening	37	40.2
Does he/she have another professional affiliation?	11	12.0
Works after shift		
No	87	94.6
Yes	4	4.3
Did not respond	1	1.1

*n = Sample

A total of 84 hours of observation were carried out, including 44 hours using multiparameter monitors, 20 hours with continuous infusion pumps and 20 hours with mechanical ventilators. In ICU A, 22 hours of observation were obtained using multiparameter monitors. In ICUs B and C, in each unit, 11 hours of observation were obtained using multiparameter monitors, 10 hours of continuous infusion pumps, and 10 hours of mechanical ventilators.

A total of 49 CAs were recorded, with a mean response time of 2.49 (± 2.8) minutes. The medical device that most frequently triggered CAs was the multiparameter monitor, with 35 records (71.4%), followed by continuous infusion pumps, with 9 records (18.4%), and mechanical ventilators, with 5 records (10.2%).

The shift with the highest frequency of triggered CAs was the morning shift (38; 77.6%). Most of the CA responses were performed by the nursing team (38; 77.5%): the professional category that responded most frequently was nursing technician (22; 44.9%), followed by nurses (16; 32.7%). Most (44; 89.8%) of the triggered CAs did not require the involvement of a second professional to resolve the altered parameter; however, of the five (10.2%) that requested assistance, three (60.0%) did so with nurses (Table 2).

Furthermore, three CAs were ignored (6.1%) during the data collection period. Of these, two (66.7%) originated from multiparameter monitors and one (33.3%) from a mechanical ventilator. The first ignored CA had a response time of 14 minutes, triggered by altered inspiratory pressure in the mechanical ventilator, attended by a nurse who subsequently contacted a physiotherapist. The second missed CA (cardiac arrest) had a response time of 5 minutes and 12 seconds, resulting from an altered heart rate, attended by a nurse who adjusted the medication administration via continuous infusion pump. Finally, the third missed CA had a response time of 13 minutes and 50 seconds, triggered by a change in blood pressure, and was attended by a nursing technician who measured the patient’s blood pressure.

Regarding the characteristics and difficulties in managing CA, among the 92 professionals, 9 (9.8%) consider the nursing staff sizing inadequate to attend to them. Of these, 7 (21.2%) were answered by nurses and 2 (3.4%) by nursing technicians. Furthermore, 37 (40.2%) professionals reported being responsible for AC configuration, with 6 (10.2%) being technicians and 31 (93.9%) being nurses; finally, one nurse (3.0%) reported difficulty in configuring AC correctly.

The majority of professionals (61; 66.3%) reported frequently or very frequently interrupting an activity to attend to CA. The activity most frequently reported as interrupted to attend to CA by nursing technicians and nurses (66; 71.7%) was providing care to other patients. It was noted that nurses are more frequently interrupted, to attend to CAs, while making entries in the patient’s chart (30; 90.9%) compared to nursing technicians (36; 61.0%) (p=0.002). In parallel, nurses (22; 66.7%) are more frequently interrupted, to attend to CAs, during case discussions with other professionals compared to nursing technicians (11; 18.6%) (p<0.001).

It was found that the perception of noise competition between CAs and non-clinical equipment was common among nurses (20; 60.6%) and nursing technicians (35; 59.3%) (p=0.981). Regarding actions taken in cases of misunderstanding of CAs from multiparameter monitors, 5 (8.5%) of nursing technicians reported contacting a doctor, while 13 (39.4%) nurses reported doing so (p<0.001). Regarding contacting a nurse, it was noted that 53 (89.8%) nursing technicians reported contacting a nurse, while 18 (54.5%) of nurses contacted their peers (p<0.001).

**Table 2:** Data related to the activation of clinical alarms in general and specialized intensive care units (n* = 49). São Paulo, SP, Brazil, 2024

Variable	n*(%)	CI^†^ 95%
Medical device		
Monitor	35 (71.4)	57.5-82.2
Continuous infusion pump	9 (18.4)	9.7-31.6
Mechanical ventilator	5 (10.2)	4.0-22.2
Occurrence shift		
Morning	38 (77.6)	64.0-87.1
Afternoon	10 (20.4)	11.3-33.8
Evening	1 (2.0)	0.0-11.7
Category of the professional who attended to the CA^‡^		
Nursing Technician	22 (44.9)	31.8-58.7
Nurse	16 (32.7)	21.1-46.7
Doctor	8 (16.3)	8.2-29.3
Physiotherapist	3 (6.1)	1.5-17.2
Parameter related to triggering		
Non-invasive blood pressure	19 (38.8)	29.7-57.8
Peripheral oxygen saturation^§^	9 (18.4)	10.9-34.7
Heart rate	7 (14.3)	7.6-29.7
End of infusion at CIP^ǁ^	3 (6.1)	1.7-18.9
Venous line occlusion in the CIP^ǁ^	2 (4.1)	0.4-16.0
Presence of an air bubble in the CIP^ǁ^	1 (2.0)	0.0-12.9
Respiratory rate in mechanical ventilation^¶^	1 (2.0)	0.0-12.9
Inspiratory pressure in mechanical ventilation^¶^	1 (2.0)	0.0-12.9
Current volume in the MV^¶^	1 (2.0)	0.0-12.9
Call in another professional	5 (10.2)	4.0-22.2
Alarms ignored	3 (6.1)	1.5-17.2

*n = Sample; ^†^CI = Confidence interval; ^‡^CA = Clinical alarm; ^§^O_2_ = Oxygen; ^ǁ^CIP = Continuous infusion pump; ^¶^MV = Mechanical ventilator

However, with regard to CAs from mechanical ventilators, 20 nursing technicians (33.9%) contacted nurses, but 100% of nurses did not request help from other nurses (p<0.001) (Table 3).

**Table 3:** Characteristics related to alarm management in general and specialized ICUs*, according to professional category (n^†^ = 92). São Paulo, SP, Brazil, 2024

Characteristics	NT^‡^	Nurse	P
	n^†^= 59	n^†^= 33	
	n^†^ (%)	n^†^ (%)	
Experienced an adverse event with a patient related to a clinical alarm	11 (18.6)	6 (18.2)	0.935
Frequent false alarms cause reduced attention or response	41 (69.5)	28 (84.8)	0.132
Difficulty understanding priority	1 (1.7)	0 (0.0)	1.000
Inadequate nursing staffing to respond to alarms	2 (3.4)	7 (21.2)	0.006
Difficulty hearing alarms	1 (1.7)	1 (3.0)	1.000
Difficulty identifying the source of an alarm	2 (3.4)	1 (3.0)	0.926
Excessive reliance on alarms to attract attention	20 (33.9)	10 (30.3)	0.726
Alarm noise competition with non-clinical equipment	35 (59.3)	20 (60.6)	0.981
Lack of training in alarm systems	22 (37.3)	9 (27.3)	0.332
Responsible for alarm configuration	6 (10.2)	31 (93.9)	<0.001
Difficulty configuring alarms correctly	0 (0.0)	1 (3.0)	1.000
Interrupts activity to respond to alarm			
Rarely	3 (5.1)	1 (3.0)	0.615
Occasionally	17 (28.8)	10 (30.3)	
Frequently	22 (37.3)	16 (48.5)	
Very frequently	17 (28.8)	6 (18.2)	
Activities interrupted to attend to CAs^§^			
Providing care to another patient	42 (71.2)	24 (72.7)	0.876
Recording information in the patient’s chart	36 (61.0)	30 (90.9)	0.002
Discussing cases with another professional	11 (18.6)	22 (66.7)	<0.001
In the patient’s own room when the CA^§^ goes off	31 (52.5)	17 (51.5)	0.925
Generally not performing any activity	1 (1.7)	1 (3.0)	1.000
There should be a dedicated person to answer the alarms			
I totally disagree	7 (11.9)	7 (21.2)	0.147
I disagree	13 (22.0)	10 (30.3)	
I neither disagree nor agree	19 (32.2)	4 (12.1)	
I agree	10 (16.9)	9 (27.3)	
I totally agree	9 (15.3)	3 (9.1)	
Receives adequate training to manage or understand alarms			
I totally disagree	1 (1.7)	0 (0.0)	0.125
I disagree	9 (15.3)	1 (3.0)	
I neither disagree nor agree	12 (20.3)	8 (24.2)	
I agree	29 (49.2)	14 (42.4)	
I totally agree	8 (13.6)	10 (30.3)	
Difficulty in understanding alarms			
I have no difficulty.	40 (67.8)	23 (69.7)	0.577
Mechanical ventilator.	9 (15.3)	8 (24.2)	
Multiparameter monitor.	3 (5.1)	9 (27.3)	
Other equipment.	3 (5.1)	2 (6.1)	
Attitude in the face of a misunderstanding of clinical alarm stemming from:			
Multiparameter monitor, only silent mode	4 (6.8)	2 (6.1)	0.894
Multiparameter monitor, alerts a doctor	5 (8.5)	13 (39.4)	<0.001
Multiparameter monitor, alerts a nurse	53 (89.8)	18 (54.5)	<0.001
Mechanical ventilator, only silent mode	1 (1.7)	0 (0.0)	1.000
Mechanical ventilator, alerts a physiotherapist	36 (61.0)	25 (75.8)	0.154
Mechanical ventilator, alerts a nurse	20 (33.9)	0 (0.0)	<0.001
Continuous infusion pump, only silent mode	1 (1.7)	0 (0.0)	1.000
Continuous infusion pump, alerts a nurse	3 (5.1)	1 (3.0)	0.645

*ICU = Intensive care unit; ^†^n = Sample; ^‡^NT= Nurse technician; ^§^CA = Clinical alarm

## Discussion

The results of this investigation pointed to the multiparameter monitor as the medical device that most frequently emitted CA. Most CAs were attended to, mainly by the nursing staff and a low frequency of ignored triggers was observed. Regarding barriers to CA management, nursing technicians and nurses considered the existence of noise competition between CA and non-clinical equipment. Inadequate staffing for CA care was prevalent in the opinion of nurses. They declared themselves responsible for configuring CA parameters and reported being more frequently interrupted by CA while making entries in the patient’s electronic medical record and discussing cases with other members of the health team compared to nursing technicians. In addition, when CA was misunderstood, nursing technicians reported to nurses.

In this study, the multiparameter monitor was responsible for emitting most CAs. These medical devices simultaneously monitor physiological oscillations, such as heart rate, respiration, blood pressure and oxygen saturation^20^. A low rate of missed CAs was observed, which differs from a study conducted in an ICU in Colombia, where just over 100 hours of CA observation were carried out and it was identified that approximately 50% of the CAs triggered were missed^21^. Although different rates have been reported, missed CAs can expose patients to significant safety threats, such as delays or non-attendance to cardiac arrhythmias, serious hyper- or hypotension events, bradypnea or apnea, for example^21^.

Noise competition between medical devices and non-clinical equipment is frequent in ICUs. In addition to making CA recognition difficult, it can impact the performance and physical and mental health of nurses, nursing technicians and patients. In addition to medical devices, noise is produced by landline phones, mobile phones and radio communicators^22^. The results of a recently published systematic literature review with meta-synthesis, which included the results of 11 studies from six countries, indicated that excessive noise in ICUs reduces the ability of nursing professionals to detect genuine CAs, which report a need of patients. In addition, it has a negative impact on their mental health, since professionals have the impression of continuing to hear the noises even after the end of their shift. The damage is also reported by patients, as it prevents rest and creates an environment that causes them fear and anxiety^23^. Thus, protocols aimed at reducing noise in ICUs and educating the health team should be implemented in order to optimize the management of CAs^22^.

The inadequate sizing of nursing staff to attend to CAs, as verified in the perception of nurses, can compromise patient safety and the quality of life of health professionals. According to Brazilian legislation, it is recommended that there be at least one nurse for up to ten patients and one technician for two patients in an ICU^24^. Therefore, inadequate nursing staffing contributes to increased workload, increased risk of accidents for workers, higher levels of mental distress among professionals, increased hospitalization costs, and reduced survival rates for patients with a higher likelihood of adverse events. Furthermore, the provision of care, including the management of adverse events, can be negatively impacted by understaffed nursing teams, leading to omissions or delays in addressing adverse events^23^ ^,^ ^25^.

Although the policy of the institution selected for this investigation indicates that the nurse is responsible for determining the parameters of CA, a portion of the nursing technician team declared that they perform this activity. This may represent a threat to patient safety, as it is the nurse’s function to act in each stage of the monitoring process, from identifying the need to executing the care plan, evaluating and recording the parameters, in order to guarantee the proper functioning of medical devices^26^. This denotes the need for ongoing in-service education, to be conducted actively so that nurses understand the objective, method and tools for promoting patient safety through CA management^20^. Added to this are the results of studies conducted in ICUs in the United States and Turkey, which together included 135 nurses in investigations on the effects of implementing an evidence-based CA management program focused on nurse training. These studies have revealed a reduction in the occurrence of false-positive CAs and a minimization of missed CA events, as well as improved nurses’ knowledge of multiparameter monitors, in addition to better adherence to evidence for CA management^27^ ^-^ ^28^.

Interruption of nursing professionals’ activities due to CAs during record keeping or case discussions with other team members can imply a lack of relevant information and threats to safety, as well as compromising continuity of care, insurance denials and even a lack of evidence in ongoing legal proceedings. The medical record has a legal, confidential and scientific character and enables communication between members of the health team^29^. The various activities performed by nurses, together with interruptions under a work overload scenario, contribute to unfinished tasks, delays and omissions in care, as well as reduced concentration and attention, increasing the risk of errors and exposing the patient to safety threats^30^ ^-^ ^31^.

Thus, although the low frequency of ignored CAs observed in this study differs from findings reported in Latin American countries, such as Colombia, it is close to the results of investigations conducted in high-income nations, in which training programs and structured alarm management protocols have contributed to reducing false-positive CAs and optimizing the response of the nursing staff. However, differences related to staff sizing, technological infrastructure, and safety culture between international contexts and Brazilian ICUs should be considered in the interpretation of these results. Thus, the findings presented here reinforce the need for investments in institutional policies and professional training focused on CA management, in order to bring national practice closer to the best internationally available evidence^23^ ^,^ ^27^.

This, to our knowledge, is the first reported national study in which CA activation data from different medical devices were collected in three ICUs comprising a significant number of beds and which analyzed the opinions of nursing professionals regarding CA management. On the other hand, the main weakness of this investigation lies in the absence of standardized instruments for assessing the barriers to alarm management in the country. In addition, the cross-sectional design prevented the monitoring of outcomes for patients exposed to ignored CAs and the fact that only approximately 50% of the nursing professionals working in the selected ICUs agreed to participate in the study may compromise the generalizability of the results found. However, the reported results remain relevant to the construction of scientific knowledge in the area.

As findings of this study, it is emphasized that the nursing team is mostly responsible for attending to CAs; even in ICUs of a hospital with a consolidated safety culture, there is an occurrence of ignored CAs and the interruption of professionals during essential nursing tasks can compromise patient safety and overload professionals. These findings may be useful for ICU nurse managers to optimize work processes, as well as to encourage researchers to undertake new studies that evaluate the outcomes of patients exposed to ignored CAs, the frequency of CAs triggered concomitantly in ICUs and their effects on the workload of the nursing staff, as well as on patient safety.

## Conclusion

Several barriers to proper management were identified, including human resources, environmental factors, and work processes. Regarding care, alarms from multiparameter monitors predominated, responded to mainly by the nursing staff, with a low proportion of ignored clinical alarms.

## Data Availability

Datasets related to this article will be available upon request to the corresponding author.
