# Supplementary material for: Barriers to managing clinical alarms in intensive care units: a cross-sectional study
Source: Rev Lat Am Enfermagem. 2026 Jun 15;34:e4843. doi: 10.1590/1518-8345.7969.4843 (PMC13277354; doi:10.1590/1518-8345.7969.4843)
Supplement: Translation

# Translation: Barreiras para o gerenciamento de alarmes clínicos em unidades de terapia intensiva: um estudo transversal

**DOI:** 10.1590/1518-8345.7969.4844

**Authors:** Renata Cristina Crippa Diekmann, Lillian Caroline Fernandes, Ramon Antonio Oliveira

**Affiliations:** 1 Sociedade Israelita Brasileira Albert Einstein, Faculdade Israelita de Ciências da Saúde Albert Einstein, São Paulo, SP, Brasil.; 2 Universidade de São Paulo, Escola de Enfermagem, São Paulo, SP, Brasil.

**Keywords:** Unidades de Terapia Intensiva, Cuidados Críticos, Segurança do Paciente, Alarmes Clínicos, Profissionais de Enfermagem, Enfermagem

## Abstract

**Objetivo:**

investigar as barreiras para o gerenciamento de alarmes clínicos segundo enfermeiros e técnicos em enfermagem e examinar características do atendimento aos alarmes clínicos em unidades de terapia intensiva.

**Método:**

estudo transversal realizado em um hospital acreditado pela *Joint Commission International* e *American Nurses Credentialing Center Magnet Recognition.* Aplicou-se formulário para identificar as barreiras para o gerenciamento dos alarmes clínicos e realizou-se 84 horas de observação.

**Resultados:**

participaram 92 profissionais (33 enfermeiros e 59 técnicos em enfermagem). A percepção de dimensionamento de pessoal de enfermagem inadequado foi mais frequente entre enfermeiros (21,2%) do que entre técnicos em enfermagem (3,4%; p=0,006). A competição entre alarmes clínicos e ruídos de outros equipamentos foi identificada como semelhante entre os enfermeiros (20; 60,6%) e técnicos em enfermagem (35; 53,9%; p=0,981). Interrupções frequentes de atividades foram relatadas por enfermeiros (6; 18,2%) e técnicos em enfermagem (17; 28,8%) (p=0,615). Registram-se 49 alarmes clínicos, com média de tempo-resposta de 2,5 minutos (DP 2,8) e três (6,1%) ignorados.

**Conclusão:**

diversas barreiras ao gerenciamento adequado foram identificadas, incluindo recursos humanos, fatores ambientais e processo de trabalho. Quanto ao atendimento, predominaram alarmes de monitores multiparâmetros, respondidos majoritariamente pela equipe de enfermagem, com baixa proporção de alarmes clínicos ignorados.

## Destaques

**(1)** O estudo investigou as barreiras para o gerenciamento de alarmes clínicos em Unidades de Terapia Intensiva. **(2)** A falta de pessoal foi apontada como barreira por 21,2% dos enfermeiros. **(3)** O ruído excessivo foi considerado um problema por 60,6% dos enfermeiros. **(4)** O ruído excessivo foi considerado uma barreira por 59,3% dos técnicos em enfermagem. **(5)** 6,1% dos alarmes clínicos foram ignorados pelos enfermeiros e técnicos em enfermagem.

## Introdução

As unidades de terapia intensiva (UTI) destinam-se ao atendimento, em sistema de vigilância contínua, de pacientes graves ou de risco, potencialmente recuperáveis, por meio do diagnóstico, tratamento e reabilitação, prestado por equipe multidisciplinar composta por médicos, enfermeiros, técnicos em enfermagem, fisioterapeutas, farmacêuticos, nutricionistas e psicólogos que promovem o cuidado centrado na pessoa^1^ ^-^ ^3^.

Para a prestação de cuidados neste cenário, é necessário o emprego de diversas tecnologias em saúde, como os dispositivos médicos^4^. Dentre eles, destacam-se monitores multiparâmetros, ventiladores mecânicos, bombas de infusão contínua e oxímetros de pulso, por exemplo. Esses dispositivos coletam dados do paciente ou de determinadas terapêuticas a ele administradas em alta frequência, ou seja, realizam a leitura da frequência cardíaca e respiratória, da saturação periférica de oxigênio, da temperatura e de condições de administração de medicamentos ou gases medicinais múltiplas vezes a cada segundo^5^. Os dispositivos médicos são considerados apoiadores de tomada de decisão clínica e, por sua relevância, são equipados com sistemas de alarmes clínicos (AC)^6^.

Os AC podem ser definidos como recursos visuais e auditivos presentes em diversos dispositivos médicos a fim de manter a segurança dos pacientes. Devem ser configurados de acordo com as características clínicas e necessidades de cada paciente, com o intuito de que sejam evitados alarmes falso-positivos, bem como eventos adversos (EA) decorrentes de situações não sinalizadas^7^. Nesse sentido, os AC são importantes ferramentas que podem contribuir para a melhoria dos padrões de segurança do paciente, pois determinados EA e incidentes que resultam em danos à saúde podem ser evitados por meio do gerenciamento dos AC emitidos pelos dispositivos médicos^8^.

A gestão de AC compreende uma combinação de intervenções baseadas em evidências, o que inclui programas educacionais, desenvolvimento de políticas e procedimentos, redução da monitorização excessiva de pacientes e personalização de parâmetros de AC^9^. Sublinhando sua importância, esse tema foi incorporado no ano de 2019 na 7ª edição do Manual Internacional de Padrões para Acreditação de Hospitais da *Joint Commission International* (JCI) e descrito no capítulo de Cuidados com os Pacientes^10^.

Diversos aspectos podem causar prejuízo à gestão de AC, como a falta de uniformidade nos padrões de monitorização, a priorização absoluta de pacientes de maior gravidade em detrimento daqueles que demandam cuidados em menor frequência ou cujas enfermidades sugerem menor gravidade, o dimensionamento dos profissionais de enfermagem inferior às necessidades e a fadiga por AC^11^. Resultados de estudos nacionais e internacionais que se dedicaram a investigar o gerenciamento de AC detiveram-se em elucidar principalmente os efeitos da fadiga por alarmes entre enfermeiros assistenciais em UTI^11^ ^-^ ^13^. Evidenciou-se que estes profissionais estão expostos aos altos níveis de fadiga por alarmes, o que ocasiona alta frequência de AC ignorados e uma relação entre fadiga por alarmes e *burnout* ^11^ ^-^ ^13^.

Nota-se, contudo, que a categoria de técnicos em enfermagem ou *nursing assistants*, que constitui parcela importante dos prestadores de cuidados de enfermagem em UTI, não tem sido incluída, o que acaba por limitar a análise do gerenciamento de AC em UTI nacionais e internacionais^11^ ^-^ ^13^. Assim, embora a literatura científica aponte efeitos deletérios da ausência do gerenciamento de AC, ainda há necessidade de investigações que propiciem uma compreensão dos fatores que afetam e dificultam o gerenciamento adequado de AC, com base nos princípios da segurança do paciente, entre os membros da equipe de enfermagem^14^.

Portanto, tomando o enunciado em consideração, este estudo teve como objetivos investigar as barreiras para o gerenciamento de alarmes clínicos segundo enfermeiros e técnicos em enfermagem e examinar características do atendimento aos alarmes clínicos em unidades de terapia intensiva.

## Método

### Delineamento do estudo

Trata-se de um estudo transversal reportado segundo as diretrizes do *Strengthening the Reporting of Observational Studies in Epidemiology* (STROBE)^15^.

### Cenário do estudo

O estudo foi realizado em três UTI de um hospital geral, privado, de grande porte, filantrópico, localizado na cidade de São Paulo, SP, Brasil e acreditado pela *Joint Commission International* e *American Nurses Credentialing Center Magnet Recognition*, com 966 leitos, entre apartamentos de clínica médica e cirúrgica, unidade semi-intensiva e unidades de terapia intensiva adulto, neonatal e pediátrica. Os 84 leitos de terapia intensiva adulto estão distribuídos em três unidades, assim designadas: UTI A, geral, com atendimento aos pacientes acometidos por agravos clínicos ou cirúrgicos; UTI B, especializada no atendimento aos pacientes acometidos por afecções neurológicas e UTI C, especializada no atendimento aos receptores de transplante de fígado.

### População e amostra

A população foi constituída por enfermeiros e técnicos em enfermagem atuantes nas UTI, de ambos os sexos; nestas unidades selecionadas para a condução da investigação, atuavam 230 enfermeiros e técnicos em enfermagem. Assim, a fim de garantir a representatividade dos resultados da investigação, foi estabelecida uma amostra não probabilística de conveniência^16^. Dos 230 profissionais acessíveis aos pesquisadores, 30 estavam afastados ou em gozo de férias. Portanto, a população foi constituída por 200 profissionais. 

### Critérios de elegibilidade

Foram incluídos enfermeiros e técnicos em enfermagem atuantes nas UTI da instituição selecionada para condução do estudo, de ambos os sexos, maiores de 18 anos de idade, independentemente do tempo de formação e de atuação, de todos os turnos e que aceitaram participar da pesquisa por meio da assinatura do Termo de Consentimento Livre e Esclarecido (TCLE).

Foram excluídos os profissionais em gozo de férias ou afastamento no período em que se deu a coleta de dados.

### Variáveis do estudo

#### Variáveis relacionadas aos profissionais

Variáveis de caracterização socioprofissional, a saber: sexo, estado civil, categoria profissional, formação em nível de especialização *lato sensu*, formação *stricto sensu*, área de atuação, turno de atuação, presença de duplo vínculo profissional e *status* de trabalho pós-plantão.

#### Variáveis relacionadas aos AC

Foram coletadas variáveis relacionadas ao acionamento dos AC e seu atendimento, a saber: dispositivo médico em que houve o acionamento do AC, turno de ocorrência do acionamento do alarme, parâmetro associado ao disparo do AC, tempo decorrido entre o acionamento do AC e seu atendimento (tempo-resposta) e ocorrência de AC ignorado. Foram considerados ignorados os AC não atendidos por um período igual ou superior a cinco minutos.

#### Variáveis e instrumento de coleta de dados relacionados às barreiras para o gerenciamento de AC

O instrumento de coleta de dados foi editado pelos autores tomando em consideração as variáveis empregadas em estudo anterior^17^ e fundamentadas nas recomendações da *Healthcare Technology Foundation* ^18^. Assim, nove variáveis foram incluídas, a saber: ocorrência de alarmes falso-positivos que levam à redução da atenção ou resposta aos AC; dificuldades em detectar ou compreender a prioridade de um AC; adequação do dimensionamento do pessoal de enfermagem para atendimento aos AC; dificuldade em ouvir AC, quando disparam; dificuldade em identificar a origem de um AC; dependência excessiva de AC para chamar atenção para os problemas dos pacientes; competição entre ruídos de AC e equipamentos não clínicos; falta de treinamento em sistemas de AC; dificuldade em configurar os AC corretamente. Para cada uma delas, solicitou-se aos profissionais de enfermagem que respondessem se observavam a sua ocorrência ou não.

### Procedimentos de gerenciamento de AC nas UTI selecionadas para condução do estudo

Na UTI A, os leitos são dispostos em apartamentos e equipados com monitores individuais; nela, a supervisão dos monitores multiparâmetros é realizada em dois pontos, no posto de enfermagem e na sala pivô. O posto de enfermagem conta com espelhamento das telas dos monitores multiparâmetros, de forma simultânea, em um monitor de vídeo e a sala pivô, um espaço físico específico localizado dentro da UTI, é equipada com dois grandes monitores de vídeo. No primeiro são espelhadas, simultaneamente, as telas dos monitores multiparâmetros de cada paciente e no segundo há exibição das imagens capturadas de cada apartamento, o que permite identificar o momento em que um profissional atende a um AC, por exemplo. Esses monitores são observados constantemente por um enfermeiro ou técnico em enfermagem. Os profissionais são capacitados para o reconhecimento de alterações nos sinais vitais, bem como em traçados eletrocardiográficos. Quando identificam alterações, notificam por telefone os membros da equipe de enfermagem responsáveis pela assistência direta aos pacientes.

Nas UTI B e C, os leitos são separados por cortinas ou por *boxes*. Nelas, os monitores multiparâmetros e demais dispositivos são observados pela equipe de enfermagem de forma direta, não havendo a supervisão dos sinais captados pelos monitores multiparâmetros por um profissional de enfermagem em uma sala pivô. Em todas as UTI, os limites mínimos e máximos configurados nos AC são determinados e ajustados diariamente, segundo as características clínicas de cada paciente, pelos enfermeiros e médicos plantonistas. Assim, de acordo com a política institucional, o enfermeiro é o profissional responsável por ajustar os parâmetros dos AC nos monitores multiparâmetros e bombas de infusão contínua das UTI.

### Coleta de dados

#### Coleta de dados dos AC

A etapa de coleta dos AC de monitores multiparâmetros, bombas de infusão contínua e ventiladores mecânicos ocorreu no período de 7 de junho a 16 de agosto de 2024 e a etapa de coleta de dados dos profissionais da enfermagem ocorreu no período de 27 de junho a 20 de setembro de 2024. 

As seguintes etapas foram percorridas: os leitos das UTI foram selecionados de forma aleatória, ou seja, houve sorteio dos números dos leitos por meio de uma lista alfanumérica previamente estabelecida pelos pesquisadores e alocada em um envelope opaco. Após o sorteio de quatro leitos, os pesquisadores registraram em um formulário de coleta o tempo-resposta do atendimento aos AC pelos profissionais com o auxílio de um cronômetro. Para aferição do tempo-resposta ao AC, optou-se por realizar a observação direta de um dispositivo médico por etapa, uma vez que não seria possível realizar este procedimento levando em consideração diversos dispositivos médicos simultaneamente. Para isso, foram realizadas 84 horas de observação, a fim de estimar a frequência de acionamento dos AC segundo cada dispositivo médico, a saber: monitor multiparâmetro, bomba de infusão contínua e ventilador mecânico.

Para o registro das razões de acionamento dos AC, do número do leito e do tempo-resposta, foram adotados os seguintes procedimentos:

- Na UTI A, os pesquisadores, um a cada turno, permaneceram na sala pivô e acompanharam o atendimento aos AC dos monitores multiparâmetros por meio dos monitores de vídeo que exibiam tanto o espelho de cada dispositivo quanto as imagens obtidas por meio das câmeras posicionadas nos apartamentos.
- Nas UTI B e C, após o sorteio dos leitos, os pesquisadores permaneceram, em todos os turnos, posicionados nos postos de enfermagem, um a cada turno, o que facilitou a visualização dos dispositivos e o atendimento aos AC.

Não foram registrados os AC acionados em decorrência de desligamento de um dos cabos para reposicionamento do paciente no leito, banho no leito, interrupção na monitorização durante a movimentação do paciente para o sanitário, deambulação e substituição de eletrodos. Não foi considerado, no processo de coleta de dados, o parâmetro de frequência respiratória, em razão de seu silenciamento por procedimento padrão, nas unidades selecionadas para condução do estudo.

#### Coleta de dados dos profissionais de enfermagem

Todos os profissionais foram convidados individualmente pelos pesquisadores, de acordo com sua disponibilidade nos postos de enfermagem de cada UTI. Para garantir ampla cobertura, a abordagem ocorreu em todos os turnos de trabalho (manhã, tarde e noite). Nesses momentos, os pesquisadores apresentavam os objetivos do estudo e formalizavam o convite à participação. Esse procedimento foi mantido até que todos os profissionais fossem contemplados.

Os profissionais foram orientados a não responder o formulário em conjunto nem discutir suas respostas com os colegas. Foi garantido anonimato em todas as etapas do estudo. Em seguida, cada participante recebeu individualmente um formulário anonimizado, contendo questões sobre características socioprofissionais e barreiras ao gerenciamento de AC. O recolhimento ocorreu em data posterior, de acordo com a preferência de cada profissional.

### Armazenamento, tratamento e análise dos dados

Após a coleta de dados em formulários físicos anonimizados, os dados foram transcritos e armazenados de forma a garantir o sigilo, a confidencialidade e a privacidade dos participantes da pesquisa no *software RedCap* ^*®*^ (Nashvillle, TN, EUA). Posteriormente, os dados foram exportados para o *software RStudio* ^*®*^ versão 1.2.5019 (RStudio, Boston, MA, EUA) e mantidos em planilha protegida por senha, sob guarda dos pesquisadores, em um microcomputador institucional.

Foram computadas as medidas de tendência central, média e desvio-padrão ou mediana e intervalo interquartil das variáveis numéricas e então foram calculadas as frequências absolutas e relativas das variáveis categóricas, com seus respectivos intervalos de confiança de 95%. Para avaliação da distribuição das variáveis numéricas, empregou-se o teste de Shapiro-Wilk e a inspeção do gráfico QQ (Quantil-Quantil). Verificou-se que as vari**á**veis numéricas apresentam distribuição diferente da normal, motivo pelo qual se utilizou o teste não paramétrico de Wilcoxon-Mann-Whitney para comparação entre grupos. A existência de associação entre duas variáveis categóricas foi verificada utilizando-se o teste de Qui-quadrado de Pearson ou, quando as tabelas de contingência apresentaram valores esperados inferiores a cinco casos, o teste Exato de Fisher.

Para todos os testes de hipóteses, utilizou-se um nível de significância de α = 5%.

### Aspectos éticos

O estudo foi aprovado pelo Comitê de Ética em Pesquisa do hospital selecionado para condução do estudo, sob o Protocolo N.º 6.800.354. Foram respeitadas as Diretrizes e Normas Regulamentadoras de Pesquisas envolvendo seres humanos, emanadas da Resolução brasileira N.º 466/12^19^ do Conselho Nacional de Saúde. 

## Resultados

Foram convidados a participar do estudo 200 profissionais de enfermagem. Destes, 92 (46,0%) participaram do estudo. Em relação à categoria profissional, 33 (35,9%) eram enfermeiros e 59 (64,1%) técnicos em enfermagem. 

A maioria dos profissionais era do sexo feminino (60; 65,2%), com mediana de idade de 34 (33,5-37,1) anos. A maioria autodeclarou-se como casada (46; 50,0%) e com filhos (49; 53,3%). A mediana do tempo de experiência profissional em UTI foi de 5 (6,0-8,1) anos. Dentre os enfermeiros, 28 eram especialistas em UTI (84,8%) e um profissional mestre em enfermagem (3,0%). Em relação ao turno de trabalho, apurou-se que 45 (48,9%) dos profissionais atuavam no período matutino, 43 (46,8%) no vespertino e 39 (42,4%) no período noturno (Tabela 1).

**Tabela 1:** Características sociodemográficas e profissionais de enfermeiros e técnicos em enfermagem que atuam em unidades de terapia intensiva geral e especializadas (n* = 92). São Paulo, SP, Brasil, 2024

Variáveis	n*	%
	(n*= 92)	
Sexo		
Masculino	30	32,6
Feminino	60	65,2
Não informado	2	2,2
Estado civil		
Solteiro	37	40,2
Casado	46	50,0
Divorciado	8	8,7
Não informado	1	1,1
Com filhos	49	53,3
Categoria profissional		
Técnico em Enfermagem	59	64,1
Enfermeiro	33	35,9
Turno em que respondeu		
Manhã	15	16,3
Tarde	40	43,5
Noite	37	40,2
Possui outro vínculo profissional	11	12,0
Trabalha após o plantão		
Não	87	94,6
Sim	4	4,3
Não respondeu	1	1,1

*n = Amostra

Foram realizadas 44 horas de observação dos monitores multiparâmetros, 20 horas de bombas de infusão contínua e 20 horas de ventiladores mecânicos, totalizando 84 horas de observação. Na UTI A, obteve-se 22 horas de observação de monitores multiparâmetros. Nas UTI B e C, em cada unidade, 11 horas de observação de monitores multiparâmetros, 10 horas de bomba de infusão contínua e 10 horas de ventiladores mecânicos. 

Foram registrados 49 AC, com a média do tempo-resposta igual a 2,49 (2,8) minutos. O dispositivo médico que mais frequentemente disparou AC foi o monitor multiparâmetros com 35 registros (71,4%), seguido da bomba de infusão contínua com 9 registros (18,4%) e o ventilador mecânico com 5 registros (10,2%). 

O turno com maior frequência de AC disparados foi o da manhã (38; 77,6%). A maioria dos atendimentos aos AC foi realizada pela equipe de enfermagem (38; 77,5%): a categoria profissional que mais os atendeu foi a de técnico em enfermagem (22; 44,9%), seguida pelos enfermeiros (16; 32,7%). A maioria (44; 89,8%) dos AC disparados não requereu o acionamento de um segundo profissional para a resolução do parâmetro alterado; porém, dos cinco (10,2%) que pediram auxílio, três (60,0%) o realizaram para enfermeiros (Tabela 2). 

Verificou-se, ademais, que três AC foram ignorados (6,1%) durante o período da coleta. Destes, dois (66,7%) eram oriundos de monitores multiparâmetros e um (33,3%) de ventilador mecânico. O primeiro AC ignorado apresentou tempo-resposta de 14 minutos, com disparo por pressão inspiratória alterada no ventilador mecânico, atendido por um enfermeiro que, na sequência, acionou um fisioterapeuta. O segundo AC ignorado teve tempo-resposta de 5 minutos e 12 segundos, decorrente de frequência cardíaca alterada, atendido por um enfermeiro, que realizou um ajuste na administração de medicamentos na bomba de infusão contínua. Por fim, o terceiro AC ignorado teve tempo-resposta de 13 minutos e 50 segundos, disparado por alteração da pressão arterial, sendo atendido por um técnico em enfermagem, que aferiu a pressão arterial do paciente.

Em relação às características e dificuldades para o gerenciamento de AC, dentre os 92 profissionais, 9 (9,8%) consideram o dimensionamento de pessoal de enfermagem inadequado para atendê-los. Desses, 7 (21,2%) foram respondidos por enfermeiros e 2 (3,4%) por técnicos em enfermagem. Ademais, 37 (40,2%) profissionais reportaram-se como responsáveis pela configuração de AC, sendo 6 (10,2%) técnicos e 31 (93,9%) enfermeiros; por fim, um enfermeiro (3,0%) relatou dificuldade em configurar os AC corretamente.

**Tabela 2:** Dados relacionados ao acionamento de alarmes clínicos em unidades de terapia intensiva geral e especializadas (n* = 49). São Paulo, SP, Brasil, 2024

Variável	n*(%)	IC^†^ 95%
Dispositivo médico		
Monitor	35 (71,4)	57,5-82,2
Bomba de infusão contínua	9 (18,4)	9,7-31,6
Ventilador mecânico	5 (10,2)	4,0-22,2
Turno de ocorrência		
Manhã	38 (77,6)	64,0-87,1
Tarde	10 (20,4)	11,3-33,8
Noite	1 (2,0)	0,0-11,7
Categoria do profissional que atendeu o AC^‡^		
Técnico em Enfermagem	22 (44,9)	31,8-58,7
Enfermeiro	16 (32,7)	21,1-46,7
Médico	8 (16,3)	8,2-29,3
Fisioterapeuta	3 (6,1)	1,5-17,2
Parâmetro relacionado ao disparo		
Pressão arterial não invasiva	19 (38,8)	29,7-57,8
Saturação periférica de O_2_^§^	9 (18,4)	10,9-34,7
Frequência cardíaca	7 (14,3)	7,6-29,7
Final da infusão na BIC^ǁ^	3 (6,1)	1,7-18,9
Oclusão de linha venosa na BIC^ǁ^	2 (4,1)	0,4-16,0
Presença de bolha de ar na BIC^ǁ^	1 (2,0)	0,0-12,9
Frequência respiratória no VM^¶^	1 (2,0)	0,0-12,9
Pressão inspiratória no VM^¶^	1 (2,0)	0,0-12,9
Volume corrente no VM^¶^	1 (2,0)	0,0-12,9
Acionamento de outro profissional	5 (10,2)	4,0-22,2
Alarmes ignorados	3 (6,1)	1,5-17,2

*n = Amostra; ^†^IC = Intervalo de confiança; ^‡^AC = Alarme clínico; ^§^O_2_ = Oxigênio; ^ǁ^BIC = Bomba de infusão contínua; ^¶^VM = Ventilador mecânico

A maioria dos profissionais (61; 66,3%) reportou interromper a realização de uma atividade para atendimento aos AC frequentemente ou muito frequentemente. A atividade interrompida para o atendimento aos AC mais frequentemente reportada por técnicos em enfermagem e enfermeiros (66; 71,7%) foi a prestação de cuidados aos outros pacientes. Notou-se que enfermeiros são mais frequentemente interrompidos, para atendimento aos AC, durante a realização de registros no prontuário do paciente (30; 90,9%) em comparação aos técnicos em enfermagem (36; 61,0%) (p=0,002). Paralelamente, enfermeiros (22; 66,7%) são mais frequentemente interrompidos, para atendimento aos AC, durante a discussão de casos com outros profissionais em comparação aos técnicos em enfermagem (11; 18,6%) (p<0,001).

Verificou-se que a percepção de competição de ruídos entre AC com equipamentos não clínicos foi comum entre enfermeiros (20; 60,6%) e técnicos em enfermagem (35; 59,3%) (p=0,981). A respeito das ações nos casos de incompreensão de AC provenientes de monitores multiparâmetros, 5 (8,5%) dos técnicos em enfermagem reportaram acionar um médico, ao passo que 13 (39,4%) enfermeiros reportaram fazê-lo (p<0,001). Em relação ao acionamento de um enfermeiro, notou-se que 53 (89,8%) técnicos em enfermagem reportaram acioná-lo, enquanto 18 (54,5%) dos enfermeiros acionaram seus pares (p<0,001). 

Entretanto, com relação aos AC provenientes de ventiladores mecânicos, 20 técnicos (33,9%) acionaram os enfermeiros, mas 100% dos enfermeiros não solicitaram ajuda de outros enfermeiros (p<0,001) (Tabela 3).

**Tabela 3:** Características relacionadas ao gerenciamento de alarmes em UTI* geral e especializadas, segundo a categoria profissional (n^†^ = 92). São Paulo, SP, Brasil, 2024

Características	TE^‡^	Enfermeiro	P
	n^†^= 59	n^†^= 33	
	n^†^ (%)	n^†^ (%)	
Vivenciou evento adverso com paciente relacionado ao alarme clínico	11 (18,6)	6 (18,2)	0,935
Alarmes falsos frequentes causam redução da atenção ou resposta	41 (69,5)	28 (84,8)	0,132
Dificuldade em compreender prioridade	1 (1,7)	0 (0,0)	1,000
Dimensionamento de pessoal de enfermagem inadequado para responder alarmes	2 (3,4)	7 (21,2)	0,006
Dificuldade em ouvir alarmes	1 (1,7)	1 (3,0)	1,000
Dificuldade em identificar a origem de alarme	2 (3,4)	1 (3,0)	0,926
Dependência excessiva de alarmes para chamar atenção	20 (33,9)	10 (30,3)	0,726
Competição de ruído de alarmes com equipamentos não clínicos	35 (59,3)	20 (60,6)	0,981
Falta de treinamento em sistemas de alarmes	22 (37,3)	9 (27,3)	0,332
Responsável pela configuração de alarmes	6 (10,2)	31 (93,9)	<0,001
Dificuldade em configurar alarmes corretamente	0 (0,0)	1 (3,0)	1,000
Interrompe atividade para atender alarme			
Raramente	3 (5,1)	1 (3,0)	0,615
Ocasionalmente	17 (28,8)	10 (30,3)	
Frequentemente	22 (37,3)	16 (48,5)	
Muito frequentemente	17 (28,8)	6 (18,2)	
Atividades interrompidas para atendimento aos AC^§^			
Prestação de cuidados a outro paciente	42 (71,2)	24 (72,7)	0,876
Registros no prontuário do paciente	36 (61,0)	30 (90,9)	0,002
Discussão de casos com outro profissional	11 (18,6)	22 (66,7)	<0,001
No quarto do próprio paciente quando AC^§^ dispara	31 (52,5)	17 (51,5)	0,925
Geralmente não está realizando nenhuma atividade	1 (1,7)	1 (3,0)	1,000
Deveria haver pessoa dedicada para atender os alarmes			
Discordo totalmente	7 (11,9)	7 (21,2)	0,147
Discordo	13 (22,0)	10 (30,3)	
Nem discordo nem concordo	19 (32,2)	4 (12,1)	
Concordo	10 (16,9)	9 (27,3)	
Concordo totalmente	9 (15,3)	3 (9,1)	
Recebe treinamento adequado para gerenciar ou compreender alarmes			
Discordo totalmente	1 (1,7)	0 (0,0)	0,125
Discordo	9 (15,3)	1 (3,0)	
Nem discordo nem concordo	12 (20,3)	8 (24,2)	
Concordo	29 (49,2)	14 (42,4)	
Concordo totalmente	8 (13,6)	10 (30,3)	
Dificuldade na compreensão de alarmes			
Não tenho nenhuma dificuldade	40 (67,8)	23 (69,7)	0,577
Ventilador mecânico	9 (15,3)	8 (24,2)	
Monitor multiparâmetros	3 (5,1)	9 (27,3)	
Outro equipamento	3 (5,1)	2 (6,1)	
Atitude na incompreensão de alarme clínico proveniente de:			
Monitor multiparâmetros, somente silencia	4 (6,8)	2 (6,1)	0,894
Monitor multiparâmetros, aciona um médico	5 (8,5)	13 (39,4)	<0,001
Monitor multiparâmetros, aciona um enfermeiro	53 (89,8)	18 (54,5)	<0,001
Ventilador mecânico, somente silencia	1 (1,7)	0 (0,0)	1,000
Ventilador mecânico, aciona fisioterapeuta	36 (61,0)	25 (75,8)	0,154
Ventilador mecânico, aciona um enfermeiro	20 (33,9)	0 (0,0)	<0,001
Bomba de infusão contínua, somente silencia	1 (1,7)	0 (0,0)	1,000
Bomba de infusão contínua, aciona um enfermeiro	3 (5,1)	1 (3,0)	0,645

*UTI = Unidade de terapia intensiva; ^†^n = Amostra; ^‡^TE = Técnico em enfermagem; ^§^AC = Alarme clínico

## Discussão

Os resultados desta investigação apontaram o monitor multiparâmetros como o dispositivo médico que mais frequentemente emitiu AC. A maioria dos AC foi atendida, sobretudo pela equipe de enfermagem e uma baixa frequência de disparos ignorados foi observada. Em relação às barreiras para o gerenciamento de AC, técnicos em enfermagem e enfermeiros consideraram a existência de competição de ruídos entre AC e equipamentos não clínicos. O dimensionamento inadequado de pessoal de enfermagem para o atendimento aos AC foi prevalente na opinião dos enfermeiros. Estes declararam-se como responsáveis pela configuração dos parâmetros de AC e reportaram ser mais frequentemente interrompidos por AC durante a realização de registros no prontuário eletrônico do paciente e discussão de casos com os demais membros da equipe de saúde, em comparação aos técnicos em enfermagem. Além disso, na vigência de incompreensão dos AC, os técnicos em enfermagem reportaram-se aos enfermeiros.

Neste estudo, o monitor multiparâmetros foi responsável pela emissão da maioria dos AC. Tais dispositivos médicos controlam simultaneamente oscilações fisiológicas, como frequência cardíaca, respiração, pressão arterial e saturação de oxigênio^20^. Uma baixa taxa de AC ignorados foi observada, o que difere de um estudo realizado em uma UTI da Colômbia, em que foram realizadas pouco mais de 100 horas de observação de AC e identificou-se que aproximadamente 50% dos AC disparados foram ignorados^21^. Embora diferentes taxas tenham sido reportadas, AC ignorados podem expor os pacientes às importantes ameaças à segurança, como atraso ou não atendimento de arritmias cardíacas, eventos de hiper ou hipotensão graves, bradipneia ou apneia, por exemplo^21^.

A competição de ruídos entre dispositivos médicos com equipamentos não clínicos é frequente nas UTI. Além de dificultarem o reconhecimento de AC, podem impactar no desempenho e na saúde física e mental de enfermeiros, técnicos em enfermagem e pacientes. Além dos dispositivos médicos, os ruídos são produzidos por telefones fixos, telefones móveis e radiocomunicadores^22^. Os resultados de uma revisão sistemática da literatura com metassíntese, recentemente publicada, que incluiu os resultados de 11 estudos provenientes de seis países apontaram que o excesso de ruídos em UTI reduz a capacidade de profissionais de enfermagem detectarem os AC genuínos, que reportam uma necessidade dos pacientes. Além disso, causa impacto negativo em sua saúde mental, uma vez que os profissionais têm a impressão de continuar ouvindo os ruídos mesmo após o término do plantão. Os danos também são reportados por pacientes, ao impedirem o descanso e criarem um ambiente que lhes causa medo e ansiedade^23^. Assim, protocolos com vistas à redução de ruído em UTI e à educação da equipe de saúde devem ser implementados com o objetivo de otimizar o gerenciamento de AC^22^.

O dimensionamento inadequado de pessoal de enfermagem para o atendimento aos AC, como verificado na percepção dos enfermeiros, pode comprometer a segurança do paciente e a qualidade de vida dos profissionais da saúde. De acordo com a legislação brasileira, recomenda-se, no mínimo, um enfermeiro para até dez pacientes e um técnico para dois pacientes em uma UTI^24^. Portanto, o insuficiente dimensionamento do pessoal de enfermagem contribui para o aumento da carga de trabalho, riscos de acidentes aos trabalhadores, elevação dos níveis de sofrimento mental dos profissionais, aumento dos custos de internação e redução de sobrevida dos pacientes com maior possibilidade de ocorrência de EA. Ademais, a prestação de cuidados, incluindo o gerenciamento de AC, pode ser negativamente impactada pelo subdimensionamento das equipes de enfermagem, com omissão ou atraso no atendimento aos AC^23^ ^,^ ^25^.

Embora a política da instituição selecionada para realização desta investigação indique o enfermeiro como responsável por determinar os parâmetros de AC, uma parcela da equipe de técnicos em enfermagem declarou realizar esta atividade. Isso pode representar ameaça à segurança do paciente, pois é função do enfermeiro atuar em cada etapa do processo de monitorização, desde a identificação da necessidade até a execução do plano de cuidados, avaliação e registro dos parâmetros, a fim de garantir o adequado funcionamento dos dispositivos médicos^26^. Isso denota a necessidade de educação permanente em serviço, a ser conduzida de maneira ativa para que os enfermeiros compreendam o objetivo, o método e as ferramentas para promoção da segurança do paciente por meio do gerenciamento de AC^20^. Somam-se a isso os resultados de estudos conduzidos em UTI nos Estados Unidos e na Turquia, que, juntos, incluíram 135 enfermeiros em investigações sobre os efeitos da implementação de um programa de gerenciamento de AC com base em evidências pautado no treinamento dos enfermeiros. Tais estudos revelaram redução na ocorrência de AC falso-positivos e minimização de eventos de AC ignorados, bem como melhora dos conhecimentos dos enfermeiros sobre monitores multiparâmetros, além de melhor adesão às evidências para o gerenciamento de AC^27^ ^-^ ^28^.

A interrupção das atividades dos profissionais de enfermagem por AC durante os registros em prontuários ou em discussões de caso com outros membros da equipe pode implicar falta de informações relevantes e ameaças à segurança, além de comprometimento da continuidade do cuidado, glosas de convênios e até mesmo falta de evidências na vigência de processos judiciais. O prontuário tem caráter legal, sigiloso e científico e possibilita a comunicação entre os membros da equipe de saúde^29^. As diversas atividades desempenhadas pelos enfermeiros, juntamente com as interrupções sob um cenário de sobrecarga de trabalho contribuem para tarefas não finalizadas, atrasos e omissões no cuidado, além da redução de concentração e atenção, aumentando o risco de erros e a exposição do paciente às ameaças para a sua segurança^30^ ^-^ ^31^.

Assim, embora a baixa frequência de AC ignorados observada neste estudo difira de achados reportados em países latino-americanos, como a Colômbia, aproxima-se de resultados de investigações conduzidas em nações de alta renda, nas quais programas de treinamento e protocolos estruturados de gerenciamento de alarmes têm contribuído para reduzir AC falso-positivos e otimizar a resposta da equipe de enfermagem. Todavia, diferenças relacionadas ao dimensionamento de pessoal, à infraestrutura tecnológica e à cultura de segurança entre os contextos internacionais e as UTI brasileiras devem ser consideradas na interpretação desses resultados. Dessa forma, os achados aqui apresentados reforçam a necessidade de investimentos em políticas institucionais e capacitação profissional voltadas ao gerenciamento de AC, a fim de aproximar a prática nacional às melhores evidências disponíveis internacionalmente^23^ ^,^ ^27^.

Este, ao nosso conhecimento, é o primeiro estudo nacional reportado em que foram coletados os dados de acionamento de AC de diferentes dispositivos médicos em três UTI que compreendem importante número de leitos e que analisou as opiniões de profissionais de enfermagem com relação ao gerenciamento de AC. Por outro lado, a principal fragilidade desta investigação repousa sobre a ausência de instrumentos padronizados para a avaliação das barreiras para o gerenciamento de alarmes no país. Acresce-se o delineamento transversal, que impediu o acompanhamento dos desfechos de pacientes expostos aos AC ignorados e o fato de que aceitaram participar do estudo aproximadamente 50% dos profissionais de enfermagem que atuam nas UTI selecionadas, o que pode comprometer a generalização dos resultados encontrados. No entanto, os resultados reportados permanecem relevantes para a construção do conhecimento científico na área.

Como achados deste estudo, sublinha-se que a equipe de enfermagem é majoritariamente responsável pelo atendimento aos AC; mesmo em UTI de um hospital com cultura de segurança consolidada, há ocorrência de AC ignorados e a interrupção dos profissionais durante tarefas de enfermagem imprescindíveis pode comprometer a segurança do paciente e a sobrecarga dos profissionais. Tais achados podem ser úteis para que enfermeiros gestores de UTI otimizem os processos de trabalho, além de propelir pesquisadores no empreendimento de novos estudos que avaliem os desfechos de pacientes expostos aos AC ignorados, a frequência de AC acionados concomitantemente em UTI e seus efeitos sobre a carga de trabalho da equipe de enfermagem, bem como sobre a segurança do paciente.

## Conclusão

Diversas barreiras ao gerenciamento adequado foram identificadas, incluindo recursos humanos, fatores ambientais e processo de trabalho. Quanto ao atendimento, predominaram alarmes de monitores multiparâmetros, respondidos majoritariamente pela equipe de enfermagem, com baixa proporção de alarmes clínicos ignorados.

## Data Availability

Os conjuntos de dados relacionados a este artigo estarão disponíveis mediante solicitação ao autor correspondente.

# Translation: Barreras para la gestión de alarmas clínicas en unidades de cuidados intensivos: un estudio transversal

**DOI:** 10.1590/1518-8345.7969.4842

**Authors:** Renata Cristina Crippa Diekmann, Lillian Caroline Fernandes, Ramon Antonio Oliveira

**Affiliations:** 1 Sociedade Israelita Brasileira Albert Einstein, Faculdade Israelita de Ciências da Saúde Albert Einstein, São Paulo, SP, Brasil.; 2 Universidade de São Paulo, Escola de Enfermagem, São Paulo, SP, Brasil.

**Keywords:** Unidades de Cuidados Intensivos, Cuidados Críticos, Seguridad del Paciente, Alarmas Clínicas, Enfermeras Practicantes, Enfermería

## Abstract

**Objetivo:**

investigar las barreras para la gestión de alarmas clínicas según enfermeros y técnicos de enfermería y examinar las características de la atención a las alarmas clínicas en unidades de cuidados intensivos.

**Método:**

estudio transversal realizado en un hospital acreditado por la *Joint Commission International* y el *American Nurses Credentialing Center Magnet Recognition*. Se aplicó un formulario para identificar las barreras para la gestión de las alarmas clínicas y se llevaron a cabo 84 horas de observación.

**Resultados:**

participaron 92 profesionales (33 enfermeros y 59 técnicos de enfermería). La percepción de dimensionamiento inadecuado del personal de enfermería fue más frecuente entre los enfermeros (21,2%) que entre los técnicos de enfermería (3,4%; p=0,006). La competencia entre alarmas clínicas y ruidos de otros equipos se identificó como similar entre los enfermeros (20; 60,6%) y los técnicos de enfermería (35; 53,9%; p=0,981). Las interrupciones frecuentes de las actividades fueron referidas por enfermeros (6; 18,2%) y técnicos de enfermería (17; 28,8%) (p=0,615). Se registraron 49 alarmas clínicas, con promedio de tiempo de respuesta de 2,5 minutos (DE 2,8) y tres (6,1%) ignoradas.

**Conclusión:**

se identificaron diversas barreras a la gestión adecuada, incluidos los recursos humanos, factores ambientales y el proceso de trabajo. En cuanto a la atención, predominaron las alarmas de monitores multiparámetros, respondidas mayoritariamente por el equipo de enfermería, con baja proporción de alarmas clínicas ignoradas.

## Destacados

**(1)** El estudio investigó las barreras para la gestión de alarmas clínicas en Unidades de Cuidados Intensivos. **(2)** La falta de personal fue señalada como barrera por 21,2% de los enfermeros. **(3)** El ruido excesivo fue considerado un problema por 60,6% de los enfermeros. **(4)** El ruido excesivo fue considerado una barrera por 59,3% de los técnicos de enfermería. **(5)** 6,1% de las alarmas clínicas fueron ignoradas por los enfermeros y los técnicos de enfermería.

## Introducción

Las unidades de terapia intensiva (UCI) se destinan a la atención, en sistema de vigilancia continua, de pacientes graves o de riesgo, potencialmente recuperables, por medio del diagnóstico, tratamiento y rehabilitación, prestados por un equipo multidisciplinar compuesto por médicos, enfermeros, técnicos de enfermería, fisioterapeutas, farmacéuticos, nutricionistas y psicólogos que promueven el cuidado centrado en la persona^1^ ^-^ ^3^.

Para la prestación de cuidados en este escenario, es necesario el empleo de diversas tecnologías en salud, como los dispositivos médicos^4^. Entre ellos, se destacan monitores multiparámetros, ventiladores mecánicos, bombas de infusión continua y oxímetros de pulso, por ejemplo. Estos dispositivos recopilan datos del paciente o de determinadas terapéuticas administradas a él con alta frecuencia, es decir, realizan la lectura de la frecuencia cardiaca y respiratoria, de la saturación periférica de oxígeno, de la temperatura y de las condiciones de administración de medicamentos o gases medicinales múltiples veces por segundo^5^. Los dispositivos médicos se consideran apoyos para la toma de decisiones clínicas y, por su relevancia, están equipados con sistemas de alarmas clínicas (AC)^6^.

Las AC pueden definirse como recursos visuales y auditivos presentes en diversos dispositivos médicos con el fin de mantener la seguridad de los pacientes. Deben configurarse de acuerdo con las características clínicas y las necesidades de cada paciente, con el objetivo de evitar alarmas falsos positivos, así como eventos adversos (EA) derivados de situaciones no señalizadas^7^. En este sentido, las AC son herramientas importantes que pueden contribuir a la mejora de los estándares de seguridad del paciente, pues determinados EA e incidentes que resultan en daños a la salud pueden evitarse mediante la gestión de las AC emitidos por los dispositivos médicos^8^.

La gestión de AC comprende una combinación de intervenciones basadas en evidencias, lo que incluye programas educativos, desarrollo de políticas y procedimientos, reducción de la monitorización excesiva de pacientes y personalización de parámetros de AC^9^. Subrayando su importancia, este tema fue incorporado en el año 2019 en la 7ª edición del Manual Internacional de Estándares para la Acreditación de Hospitales de la *Joint Commission International* (JCI), descrito en el capítulo de Cuidados del Paciente^10^.

Diversos aspectos pueden perjudicar la gestión de AC, como la falta de uniformidad en los estándares de monitorización, la priorización absoluta de pacientes de mayor gravedad en detrimento de aquellos que demandan cuidados con menor frecuencia o cuyas enfermedades sugieren menor gravedad, el dimensionamiento de los profesionales de enfermería por debajo de las necesidades y la fatiga por AC^11^. Resultados de estudios nacionales e internacionales dedicados a investigar la gestión de AC se centraron en elucidar principalmente los efectos de la fatiga por alarmas entre enfermeros asistenciales en UCI^11^ ^-^ ^13^. Se evidenció que estos profesionales están expuestos a altos niveles de fatiga por alarmas, lo que ocasiona alta frecuencia de AC ignoradas y una relación entre la fatiga por alarmas y el *burnout* ^11^ ^-^ ^13^.

Se observa, sin embargo, que la categoría de técnicos de enfermería o *nursing assistants*, que constituye una parte importante de los proveedores de cuidados de enfermería en UCI, no ha sido incluida, lo que termina por limitar el análisis de la gestión de AC en UCI nacionales e internacionales^11^ ^-^ ^13^. Así, aunque la literatura científica señale efectos deletéreos de la ausencia de gestión de AC, aún existe la necesidad de investigaciones que proporcionen una comprensión de los factores que afectan y dificultan la gestión adecuada de AC, con base en los principios de la seguridad del paciente, entre los miembros del equipo de enfermería^14^.

Por lo tanto, tomando en consideración lo expuesto, este estudio tuvo como objetivos investigar las barreras para la gestión de alarmas clínicas según enfermeros y técnicos de enfermería y examinar las características de la atención a las alarmas clínicas en unidades de terapia intensiva.

## Método

### Diseño del estudio

Se trata de un estudio transversal reportado según las directrices del *Strengthening the Reporting of Observational Studies in Epidemiology* (STROBE)^15^.

### Escenario del estudio

El estudio fue realizado en tres UCI de un hospital general, privado, de gran tamaño, filantrópico, ubicado en la ciudad de São Paulo, SP, Brasil y acreditado por la *Joint Commission International* y el *American Nurses Credentialing Center Magnet Recognition*, con 966 camas, entre habitaciones de clínica médica y quirúrgica, unidad de cuidados semiintensivos y unidades de cuidados intensivos de adultos, neonatales y pediátricas. Las 84 camas de cuidados intensivos de adultos están distribuidas en tres unidades, de la siguiente manera: UCI A, general, que atiende a pacientes con afecciones clínicas o quirúrgicas; UCI B, especializada en la atención de pacientes con afecciones neurológicas; y UCI C, especializada en la atención de receptores de trasplante de hígado. 

### Población y muestra

La población estuvo constituida por enfermeras y técnicos de enfermería que actuaban en las UCI, de ambos sexos; en estas unidades seleccionadas para la realización de la investigación se desempeñaban 230 enfermeras y técnicos de enfermería. Así, con el fin de garantizar la representatividad de los resultados de la investigación, se estableció una muestra no probabilística por conveniencia^16^. De los 230 profesionales accesibles a los investigadores, 30 se encontraban de baja o de vacaciones. Por lo tanto, la población estuvo constituida por 200 profesionales.

### Criterios de elegibilidad

Se incluyeron enfermeros y técnicos de enfermería que actuaban en las UCI de la institución seleccionada para la realización del estudio, de ambos sexos, mayores de 18 años, independientemente del tiempo de formación y de experiencia profesional, de todos los turnos de trabajo y que aceptaron participar en la investigación mediante la firma del Consentimiento Informado (CI).

Se excluyeron los profesionales que estaban de vacaciones o de baja en el período en que se realizó la recogida de datos.

### Variables del estudio

#### Variables relacionadas con los profesionales

Variables de caracterización socioprofesional, a saber: sexo, estado civil, categoría profesional, formación a nivel de especialización *lato sensu*, formación *stricto sensu*, área de actuación, turno de trabajo, presencia de doble vínculo profesional y situación laboral posguardia.

#### Variables relacionadas con los AC

Se recogieron variables relacionadas con la activación de las AC y su atención: dispositivo médico en el que se produjo la activación de la AC, turno en el que ocurrió la activación de la alarma, parámetro asociado al disparo de la AC, tiempo transcurrido entre la activación de la AC y su atención (tiempo de respuesta) y ocurrencia de AC ignorada. Se consideraron ignorados las AC no atendidas durante un período igual o superior a cinco minutos.

#### Variables e instrumento de recogida de datos relacionados con las barreras para la gestión de AC

El instrumento de recogida de datos fue elaborado por los autores teniendo en cuenta las variables empleadas en un estudio previo^17^ y fundamentadas en las recomendaciones de la *Healthcare Technology Foundation* ^18^. Así, se incluyeron nueve variables: ocurrencia de alarmas falsos positivos que llevan a la reducción de la atención o respuesta a las AC; dificultades para detectar o comprender la prioridad de una AC; adecuación del dimensionamiento del personal de enfermería para la atención de las AC; dificultad para oír las AC cuando se activan; dificultad para identificar el origen de una AC; dependencia excesiva de las AC para llamar la atención sobre los problemas de los pacientes; competencia entre ruidos de AC y equipos no clínicos; falta de capacitación en sistemas de AC; dificultad para configurar correctamente las AC. Para cada una de ellas, se solicitó a los profesionales de enfermería que respondieran si observaban su ocurrencia o no.

### Procedimientos de gestión de AC en las UCI seleccionadas para la realización del estudio

En la UCI A, las camas se disponen en habitaciones individuales y están equipadas con monitores individuales; en ella, la supervisión de los monitores multiparámetros se realiza en dos puntos: en el puesto de enfermería y en la sala de monitoreo central. El puesto de enfermería cuenta con el reflejo simultáneo de las pantallas de los monitores multiparámetros en un monitor de vídeo y la sala de monitoreo central, un espacio físico específico ubicado dentro de la UCI, está equipada con dos grandes monitores de vídeo. En el primero se reflejan, simultáneamente, las pantallas de los monitores multiparámetros de cada paciente y en el segundo se muestran las imágenes captadas de cada habitación, lo que permite identificar el momento en que un profesional atiende a una AC, por ejemplo. Estos monitores son observados constantemente por un enfermero o un técnico de enfermería. Los profesionales están capacitados para el reconocimiento de alteraciones en los signos vitales, así como en los trazados electrocardiográficos. Cuando identifican alteraciones, notifican por teléfono a los miembros del equipo de enfermería responsables de la atención directa a los pacientes.

En las UCI B y C, las camas están separadas por cortinas o por boxes. En ellas, los monitores multiparámetros y los demás dispositivos son observados directamente por el equipo de enfermería, no habiendo supervisión de las señales captadas por los monitores multiparámetros por parte de un profesional de enfermería en una sala de monitoreo central. En todas las UCI, los límites mínimos y máximos configurados en las AC son determinados y ajustados diariamente, según las características clínicas de cada paciente, por los enfermeros y los médicos de guardia. Así, de acuerdo con la política institucional, el enfermero es el profesional responsable de ajustar los parámetros de las AC en los monitores multiparámetros y en las bombas de infusión continua de las UCI.

### Recogida de datos

#### Recogida de datos de los AC

La fase de recogida de las AC de monitores multiparámetros, bombas de infusión continua y ventiladores mecánicos tuvo lugar en el período del 7 de junio al 16 de agosto de 2024 y la fase de recogida de datos de los profesionales de enfermería se realizó del 27 de junio al 20 de septiembre de 2024.

Se llevaron a cabo las siguientes etapas: las camas de las UCI fueron seleccionadas de forma aleatoria, es decir, se sortearon los números de las camas por medio de una lista alfanumérica previamente elaborada por los investigadores y colocada en un sobre opaco. Tras el sorteo de cuatro camas, los investigadores registraron en un formulario de recogida el tiempo de respuesta de la atención a las AC por parte de los profesionales con la ayuda de un cronómetro. Para la medición del tiempo de respuesta a la AC, se optó por realizar la observación directa de un dispositivo médico por etapa, ya que no sería posible realizar este procedimiento teniendo en cuenta varios dispositivos médicos de forma simultánea. Para ello, se llevaron a cabo 84 horas de observación, con el fin de estimar la frecuencia de activación de las AC según cada dispositivo médico: monitor multiparámetro, bomba de infusión continua y ventilador mecánico.

Para el registro de las razones de activación de las AC, del número de la cama y del tiempo de respuesta, se adoptaron los siguientes procedimientos:

- En la UCI A, los investigadores, uno en cada turno, permanecieron en la sala de monitoreo central y acompañaron la atención a las AC de los monitores multiparámetros por medio de los monitores de vídeo que mostraban tanto el espejo de cada dispositivo como las imágenes obtenidas por las cámaras posicionadas en las habitaciones.
- En las UCI B y C, tras el sorteo de las camas, los investigadores permanecieron, en todos los turnos, ubicados en los puestos de enfermería, uno en cada turno, lo que facilitó la visualización de los dispositivos y la atención a las AC.

No se registraron las AC activadas como consecuencia de la desconexión de alguno de los cables para el reposicionamiento del paciente en la cama, baño en la cama, interrupción de la monitorización durante el traslado del paciente al baño, deambulación y sustitución de electrodos. No se tuvo en cuenta, en el proceso de recogida de datos, el parámetro de frecuencia respiratoria, debido a su silenciamiento por procedimiento estándar en las unidades seleccionadas para la realización del estudio.

#### Recogida de datos de los profesionales de enfermería

Todos los profesionales fueron invitados individualmente por los investigadores, de acuerdo con su disponibilidad en los puestos de enfermería de cada UCI. Para garantizar una cobertura amplia, el abordaje se realizó en todos los turnos de trabajo (mañana, tarde y noche). En esos momentos, los investigadores presentaban los objetivos del estudio y formalizaban la invitación a participar. Este procedimiento se mantuvo hasta que todos los profesionales fueran contemplados.

Los profesionales fueron orientados a no responder el formulario en conjunto ni discutir sus respuestas con los colegas. Se garantizó el anonimato en todas las etapas del estudio. A continuación, cada participante recibió individualmente un formulario anonimizado, que contenía preguntas sobre características socioprofesionales y barreras para la gestión de las AC. La recogida de los formularios se realizó en una fecha posterior, de acuerdo con la preferencia de cada profesional.

### Almacenamiento, tratamiento y análisis de los datos

Tras la recogida de datos en formularios físicos anonimizados, los datos fueron transcritos y almacenados de forma que se garantizara el sigilo, la confidencialidad y la privacidad de los participantes de la investigación en el *software* RedCap^®^ (Nashvillle, TN, EE. UU.). Posteriormente, los datos fueron exportados al *software* RStudio^®^ versión 1.2.5019 (RStudio, Boston, MA, EE. UU.) y se mantuvieron en una hoja de cálculo protegida por contraseña, bajo custodia de los investigadores, en una computadora institucional.

Se calcularon las medidas de tendencia central, media y desviación estándar o mediana e intervalo intercuartílico de las variables numéricas y luego se calcularon las frecuencias absolutas y relativas de las variables categóricas, con sus respectivos intervalos de confianza del 95%. Para la evaluación de la distribución de las variables numéricas se empleó la prueba de Shapiro-Wilk y la inspección del gráfico Q-Q (Cuantil-Cuantil). Se verificó que las variables numéricas presentaban una distribución distinta de la normal, motivo por el cual se utilizó la prueba no paramétrica de Wilcoxon-Mann-Whitney para la comparación entre grupos. La existencia de asociación entre dos variables categóricas se verificó mediante la prueba de chi-cuadrado de Pearson o, cuando las tablas de contingencia presentaron valores esperados inferiores a cinco casos, la prueba exacta de Fisher.

Para todas las pruebas de hipótesis se utilizó un nivel de significancia de α = 5%.

### Aspectos éticos

El estudio fue aprobado por el Comité de Ética en Investigación del hospital seleccionado para la realización del estudio, bajo el Protocolo n.º 6.800.354. Se respetaron las Directrices y Normas Reguladoras de Investigaciones que involucran seres humanos, emanadas de la Resolución Brasileña n.º 466/12^19^ del *Conselho Nacional de Saúde*. 

## Resultados

Fueron invitados a participar en el estudio 200 profesionales de enfermería. De ellos, 92 (46,0%) participaron en el estudio. En relación con la categoría profesional, 33 (35,9%) eran enfermeros y 59 (64,1%) técnicos de enfermería.

La mayoría de los profesionales era de sexo femenino (60; 65,2%), con una mediana de edad de 34 (33,5-37,1) años. La mayoría se autodeclaró casada (46; 50,0%) y con hijos (49; 53,3%). La mediana del tiempo de experiencia profesional en UCI fue de 5 (6,0-8,1) años. Entre los enfermeros, 28 eran especialistas en UCI (84,8%) y un profesional tenía título de magíster en enfermería (3,0%). En relación con el turno de trabajo, se constató que 45 (48,9%) de los profesionales actuaban en el turno matutino, 43 (46,8%) en el turno vespertino y 39 (42,4%) en el turno nocturno (Tabla 1).

**Tabla 1:** Características sociodemográficas y profesionales de enfermeras y técnicos de enfermería que actúan en unidades de cuidados intensivos generales y especializadas (n* = 92). São Paulo, SP, Brasil, 2024

Variables	n*	%
	(n*= 92)	
Sexo		
Masculino	30	32,6
Femenino	60	65,2
No informado	2	2,2
Estado civil		
Soltero	37	40,2
Casado	46	50,0
Divorciado	8	8,7
No informado	1	1,1
Con hijos	49	53,3
Categoría profesional		
Técnico de enfermería	59	64,1
Enfermero	33	35,9
Turno en que respondió		
Mañana	15	16,3
Tarde	40	43,5
Noche	37	40,2
Tiene otro vínculo profesional	11	12,0
Trabaja después de la guardia		
No	87	94,6
Sí	4	4,3
No respondió	1	1,1

*n = Muestra

Se realizaron 44 horas de observación de los monitores multiparámetros, 20 horas de bombas de infusión continua y 20 horas de ventiladores mecánicos, totalizando 84 horas de observación. En la UCI A, se obtuvieron 22 horas de observación de monitores multiparámetros. En las UCI B y C, en cada unidad, 11 horas de observación de monitores multiparámetros, 10 horas de bombas de infusión continua y 10 horas de ventiladores mecánicos.

Se registraron 49 AC, con una media del tiempo de respuesta igual a 2,49 (2,8) minutos. El dispositivo médico que con mayor frecuencia activó AC fue el monitor multiparámetro, con 35 registros (71,4%), seguido de la bomba de infusión continua con 9 registros (18,4%) y el ventilador mecánico con 5 registros (10,2%).

El turno con mayor frecuencia de AC activadas fue el de la mañana (38; 77,6%). La mayoría de las atenciones a las AC fue realizada por el equipo de enfermería (38; 77,5%): la categoría profesional que más los atendió fue la de técnico de enfermería (22; 44,9%), seguida de los enfermeros (16; 32,7%). La mayoría (44; 89,8%) de las AC activadas no requirió la intervención de un segundo profesional para la resolución del parámetro alterado; sin embargo, de los cinco (10,2%) que solicitaron ayuda, tres (60,0%) lo hicieron hacia enfermeras (Tabla 2).

Se verificó, además, que tres AC fueron ignoradas (6,1%) durante el período de recogida. De ellos, dos (66,7%) procedían de monitores multiparámetros y uno (33,3%) de ventilador mecánico. La primera AC ignorada presentó un tiempo de respuesta de 14 minutos, con activación por presión inspiratoria alterada en el ventilador mecánico, atendido por una enfermera que, a continuación, solicitó la intervención de un fisioterapeuta. La segunda AC ignorada tuvo un tiempo de respuesta de 5 minutos y 12 segundos, debido a frecuencia cardiaca alterada, atendido por una enfermera, que realizó un ajuste en la administración de medicamentos en la bomba de infusión continua. Por último, la tercera AC ignorada tuvo un tiempo de respuesta de 13 minutos y 50 segundos, activada por alteración de la presión arterial, siendo atendido por un técnico de enfermería, que midió la presión arterial del paciente.

**Tabla 2:** Datos relacionados con la activación de alarmas clínicas en unidades de cuidados intensivos generales y especializadas (n* = 49). São Paulo, SP, Brasil, 2024

Variable	n*(%)	IC^†^ 95%
Dispositivo médico		
Monitor	35 (71,4)	57,5-82,2
Bomba de infusión continua	9 (18,4)	9,7-31,6
Ventilador mecánico	5 (10,2)	4,0-22,2
Turno de ocurrencia		
Mañana	38 (77,6)	64,0-87,1
Tarde	10 (20,4)	11,3-33,8
Noche	1 (2,0)	0,0-11,7
Categoría del profesional que atendió la AC^‡^		
Técnico de enfermería	22 (44,9)	31,8-58,7
Enfermero	16 (32,7)	21,1-46,7
Médico	8 (16,3)	8,2-29,3
Fisioterapeuta	3 (6,1)	1,5-17,2
Parámetro relacionado con la activación		
Presión arterial no invasiva	19 (38,8)	29,7-57,8
Saturación periférica de O_2_^§^	9 (18,4)	10,9-34,7
Frecuencia cardiaca	7 (14,3)	7,6-29,7
Final de la infusión en la BIC^ǁ^	3 (6,1)	1,7-18,9
Oclusión de la línea venosa en la BIC^ǁ^	2 (4,1)	0,4-16,0
Presencia de burbuja de aire en la BIC^ǁ^	1 (2,0)	0,0-12,9
Frecuencia respiratoria en el VM^¶^	1 (2,0)	0,0-12,9
Presión inspiratoria en el VM^¶^	1 (2,0)	0,0-12,9
Volumen corriente en el VM^¶^	1 (2,0)	0,0-12,9
Activación de otro profesional	5 (10,2)	4,0-22,2
Alarmas ignoradas	3 (6,1)	1,5-17,2

*n = Muestra; ^†^IC = Intervalo de confianza; ^‡^AC = Alarma clínica; ^§^O2 = Oxígeno; ^ǁ^BIC = Bomba de infusión continua; ^¶^VM = Ventilador mecánico

En relación con las características y dificultades para la gestión de AC, entre los 92 profesionales, 9 (9,8%) consideran inadecuado el dimensionamiento del personal de enfermería para atenderlos. De ellos, 7 (21,2%) fueron respuestas de enfermeras y 2 (3,4%) de técnicos de enfermería. Además, 37 (40,2%) profesionales se declararon responsables de la configuración de las AC, siendo 6 (10,2%) técnicos y 31 (93,9%) enfermeras; por último, una enfermera (3,0%) informó dificultad para configurar correctamente las AC.

La mayoría de los profesionales (61; 66,3%) refirió interrumpir la realización de una actividad para atender a las AC con frecuencia o con mucha frecuencia. La actividad interrumpida para la atención a las AC más frecuentemente reportada por técnicos de enfermería y enfermeros (66; 71,7%) fue la prestación de cuidados a otros pacientes. Se observó que los enfermeros son interrumpidos con mayor frecuencia, para la atención a las AC, durante la realización de registros en la historia clínica del paciente (30; 90,9%) en comparación con los técnicos de enfermería (36; 61,0%) (p=0,002). Paralelamente, los enfermeros (22; 66,7%) son interrumpidas con mayor frecuencia, para la atención a las AC, durante la discusión de casos con otros profesionales en comparación con los técnicos de enfermería (11; 18,6%) (p<0,001).

Se verificó que la percepción de competencia de ruidos entre AC y equipos no clínicos fue común entre enfermeros (20; 60,6%) y técnicos de enfermería (35; 59,3%) (p=0,981). Con respecto a las acciones en los casos de incomprensión de AC procedentes de monitores multiparámetros, 5 (8,5%) de los técnicos de enfermería informaron que llamaban a un médico, mientras que 13 (39,4%) enfermeros informaron hacerlo (p<0,001). En relación con la solicitud de intervención de uno enfermero, se observó que 53 (89,8%) técnicos de enfermería informaron que lo solicitaban, mientras que 18 (54,5%) de los enfermeros solicitaron a sus pares (p<0,001).

Sin embargo, en relación con las AC procedentes de ventiladores mecánicos, 20 técnicos (33,9%) solicitaron la intervención de enfermeros, pero 100% de los enfermeros no solicitaron ayuda de otros enfermeros (p<0,001) (Tabla 3).

**Tabla 3:** Características relacionadas con la gestión de alarmas en UCI* generales y especializadas, según la categoría profesional (n^†^ = 92). São Paulo, SP, Brasil, 2024

Características	TE^‡^	Enfermero	P
	n^†^= 59	n^†^= 33	
	n^†^ (%)	n^†^ (%)	
Vivenció evento adverso con paciente relacionado con alarma clínica	11 (18,6)	6 (18,2)	0,935
Alarmas falsas frecuentes provocan reducción de la atención o respuesta	41 (69,5)	28 (84,8)	0,132
Dificultad para comprender la prioridad	1 (1,7)	0 (0,0)	1,000
Dimensionamiento del personal de enfermería inadecuado para responder a las alarmas	2 (3,4)	7 (21,2)	0,006
Dificultad para oír las alarmas	1 (1,7)	1 (3,0)	1,000
Dificultad para identificar el origen de la alarma	2 (3,4)	1 (3,0)	0,926
Dependencia excesiva de las alarmas para llamar la atención	20 (33,9)	10 (30,3)	0,726
Competencia de ruido de alarmas con equipos no clínicos	35 (59,3)	20 (60,6)	0,981
Falta de capacitación en sistemas de alarmas	22 (37,3)	9 (27,3)	0,332
Responsable de la configuración de alarmas	6 (10,2)	31 (93,9)	<0,001
Dificultad para configurar correctamente las alarmas	0 (0,0)	1 (3,0)	1,000
Interrumpe actividad para atender la alarma			
Raramente	3 (5,1)	1 (3,0)	0,615
Ocasionalmente	17 (28,8)	10 (30,3)	
Frecuentemente	22 (37,3)	16 (48,5)	
Muy frecuentemente	17 (28,8)	6 (18,2)	
Actividades interrumpidas para la atención de las AC^§^			
Prestación de cuidados a otro paciente	42 (71,2)	24 (72,7)	0,876
Registros en la historia clínica del paciente	36 (61,0)	30 (90,9)	0,002
Discusión de casos con otro profesional	11 (18,6)	22 (66,7)	<0,001
En la habitación del propio paciente cuando la AC^§^ se activa	31 (52,5)	17 (51,5)	0,925
Generalmente no está realizando ninguna actividad	1 (1,7)	1 (3,0)	1,000
Debería haber una persona dedicada a atender las alarmas			
Estoy totalmente en desacuerdo	7 (11,9)	7 (21,2)	0,147
Estoy en desacuerdo	13 (22,0)	10 (30,3)	
Ni estoy de acuerdo ni en desacuerdo	19 (32,2)	4 (12,1)	
Estoy de acuerdo	10 (16,9)	9 (27,3)	
Estoy totalmente de acuerdo	9 (15,3)	3 (9,1)	
Recibe capacitación adecuada para gestionar o comprender las alarmas			
Estoy totalmente en desacuerdo	1 (1,7)	0 (0,0)	0,125
Estoy en desacuerdo	9 (15,3)	1 (3,0)	
Ni estoy de acuerdo ni en desacuerdo	12 (20,3)	8 (24,2)	
Estoy de acuerdo	29 (49,2)	14 (42,4)	
Estoy totalmente de acuerdo	8 (13,6)	10 (30,3)	
Dificultad en la comprensión de las alarmas			
No tengo ninguna dificultad	40 (67,8)	23 (69,7)	0,577
Ventilador mecánico	9 (15,3)	8 (24,2)	
Monitor multiparámetros	3 (5,1)	9 (27,3)	
Otro equipo	3 (5,1)	2 (6,1)	
Actitud ante la incomprensión de una alarma clínica procedente de:			
Monitor multiparámetros, solamente lo silencia	4 (6,8)	2 (6,1)	0,894
Monitor multiparámetros, llama a un médico	5 (8,5)	13 (39,4)	<0,001
Monitor multiparámetros, llama a un enfermero	53 (89,8)	18 (54,5)	<0,001
Ventilador mecánico, solamente lo silencia	1 (1,7)	0 (0,0)	1,000
Ventilador mecánico, llama a un fisioterapeuta	36 (61,0)	25 (75,8)	0,154
Ventilador mecánico, llama a un enfermero	20 (33,9)	0 (0,0)	<0,001
Bomba de infusión continua, solamente la silencia	1 (1,7)	0 (0,0)	1,000
Bomba de infusión continua, llama a un enfermero	3 (5,1)	1 (3,0)	0,645

*UCI = Unidad de cuidados intensivos; ^†^n = Muestra; ^‡^TE = Técnico de enfermería; ^§^AC = Alarma clínica

## Discusión

Los resultados de esta investigación señalaron al monitor multiparámetro como el dispositivo médico que con mayor frecuencia emitió AC. La mayoría de las AC fue atendida, sobre todo por el equipo de enfermería y se observó una baja frecuencia de activaciones ignoradas. En relación con las barreras para la gestión de AC, técnicos de enfermería y enfermeros consideraron la existencia de competencia de ruidos entre AC y equipos no clínicos. El dimensionamiento inadecuado del personal de enfermería para la atención de las AC fue prevalente en la opinión de los enfermeros. Estos se declararon responsables de la configuración de los parámetros de AC y refirieron ser interrumpidas con mayor frecuencia por AC durante la realización de registros en la historia clínica electrónica del paciente y la discusión de casos con los demás miembros del equipo de salud, en comparación con los técnicos de enfermería. Además, ante la incomprensión de las AC, los técnicos de enfermería recurrieron a los enfermeros.

En este estudio, el monitor multiparámetro fue responsable de la emisión de la mayoría de las AC. Dichos dispositivos médicos controlan simultáneamente oscilaciones fisiológicas, como frecuencia cardiaca, respiración, presión arterial y saturación de oxígeno^20^. Se observó una baja tasa de AC ignoradas, lo que difiere de un estudio realizado en una UCI de Colombia, en el que se llevaron a cabo poco más de 100 horas de observación de AC y se identificó que aproximadamente 50% de las AC activadas fueron ignoradas^21^. Aunque se hayan reportado tasas diferentes, las AC ignoradas pueden exponer a los pacientes a importantes amenazas para la seguridad, como retraso o ausencia de atención a arritmias cardiacas, eventos de hiper o hipotensión graves, bradipnea o apnea, por ejemplo^21^.

La competencia de ruidos entre dispositivos médicos y equipos no clínicos es frecuente en las UCI. Además de dificultar el reconocimiento de las AC, puede repercutir en el desempeño y en la salud física y mental de los enfermeros, técnicos de enfermería y pacientes. Además de los dispositivos médicos, los ruidos son producidos por teléfonos fijos, teléfonos móviles y radiocomunicadores^22^. Los resultados de una revisión sistemática de la literatura con metasíntesis, publicada recientemente, que incluyó los resultados de 11 estudios procedentes de seis países, señalaron que el exceso de ruidos en UCI reduce la capacidad de los profesionales de enfermería para detectar las AC genuinas, que señalan una necesidad de los pacientes. Además, causa un impacto negativo en su salud mental, ya que los profesionales tienen la sensación de seguir oyendo los ruidos incluso después de finalizar la guardia. Los daños también son referidos por los pacientes, al impedir el descanso y crear un entorno que les provoca miedo y ansiedad^23^. Así, deben implementarse protocolos orientados a la reducción del ruido en UCI y a la educación del equipo de salud, con el objetivo de optimizar la gestión de AC^22^.

El dimensionamiento inadecuado del personal de enfermería para la atención de las AC, tal como se verificó en la percepción de los enfermeros, puede comprometer la seguridad del paciente y la calidad de vida de los profesionales de la salud. De acuerdo con la legislación brasileña, se recomienda, como mínimo, un enfermero para hasta diez pacientes y un técnico para dos pacientes en una UCI^24^. Por lo tanto, el insuficiente dimensionamiento del personal de enfermería contribuye al aumento de la carga de trabajo, a los riesgos de accidentes laborales, a la elevación de los niveles de sufrimiento mental de los profesionales, al aumento de los costos de hospitalización y a la reducción de la supervivencia de los pacientes, con mayor probabilidad de ocurrencia de EA. Además, la prestación de cuidados, incluida la gestión de AC, puede verse negativamente afectada por el subdimensionamiento de los equipos de enfermería, con omisión o retraso en la atención a las AC^23^ ^,^ ^25^.

Aunque la política de la institución seleccionada para la realización de esta investigación indique a lo enfermero como responsable de determinar los parámetros de AC, una parte del equipo de técnicos de enfermería declaró realizar esta actividad. Esto puede representar una amenaza para la seguridad del paciente, pues es función de lo enfermero actuar en cada etapa del proceso de monitorización, desde la identificación de la necesidad hasta la ejecución del plan de cuidados, la evaluación y el registro de los parámetros, a fin de garantizar el adecuado funcionamiento de los dispositivos médicos^26^. Esto denota la necesidad de educación permanente en el servicio, que debe ser conducida de manera activa para que los enfermeros comprendan el objetivo, el método y las herramientas para promover la seguridad del paciente por medio de la gestión de AC^20^. A ello se suman los resultados de estudios realizados en UCI de Estados Unidos y Turquía que, en conjunto, incluyeron a 135 enfermeros en investigaciones sobre los efectos de la implementación de un programa de gestión de AC basado en evidencias y centrado en la capacitación de los enfermeros. Tales estudios revelaron una reducción en la ocurrencia de AC falsas positivas y la minimización de eventos de AC ignoradas, así como una mejora en los conocimientos de los enfermeros sobre monitores multiparámetros, además de una mejor adhesión a las evidencias para la gestión de AC^27^ ^-^ ^28^.

La interrupción de las actividades de los profesionales de enfermería por AC durante los registros en historias clínicas o en discusiones de casos con otros miembros del equipo puede implicar falta de informaciones relevantes y amenazas para la seguridad, además de comprometer la continuidad del cuidado, generar glosas de convenios e incluso la ausencia de evidencias en la vigencia de procesos judiciales. La historia clínica tiene carácter legal, confidencial y científico y posibilita la comunicación entre los miembros del equipo de salud^29^. Las diversas actividades desempeñadas por los enfermeros, junto con las interrupciones en un escenario de sobrecarga de trabajo, contribuyen a tareas no finalizadas, retrasos y omisiones en el cuidado, además de la reducción de la concentración y la atención, aumentando el riesgo de errores y la exposición del paciente a amenazas para la seguridad^30^ ^-^ ^31^.

Así, aunque la baja frecuencia de AC ignoradas observada en este estudio difiere de hallazgos reportados en países latinoamericanos, como Colombia, se aproxima a los resultados de investigaciones realizadas en naciones de altos ingresos, en las cuales programas de capacitación y protocolos estructurados de gestión de alarmas han contribuido a reducir AC falsas positivas y a optimizar la respuesta del equipo de enfermería. Sin embargo, deben considerarse, en la interpretación de estos resultados, las diferencias relacionadas con el dimensionamiento de personal, la infraestructura tecnológica y la cultura de seguridad entre los contextos internacionales y las UCI brasileñas. De este modo, los hallazgos aquí presentados refuerzan la necesidad de inversiones en políticas institucionales y en capacitación profesional orientadas a la gestión de AC, con el fin de aproximar la práctica nacional a las mejores evidencias disponibles internacionalmente^23^ ^,^ ^27^.

Este es, según nuestro conocimiento, el primer estudio nacional reportado en el que se recopilaron datos de activación de AC procedentes de diferentes dispositivos médicos en tres UCI que comprenden un número importante de camas y que analizó las opiniones de profesionales de enfermería en relación con la gestión de AC. Por otro lado, la principal debilidad de esta investigación radica en la ausencia de instrumentos estandarizados para la evaluación de las barreras para la gestión de alarmas en el país. Se suma el diseño transversal, que impidió el seguimiento de los desenlaces de pacientes expuestos a AC ignoradas y el hecho de que aproximadamente 50% de los profesionales de enfermería que actúan en las UCI seleccionadas aceptaron participar en el estudio, lo que puede comprometer la generalización de los resultados encontrados. No obstante, los resultados reportados siguen siendo relevantes para la construcción del conocimiento científico en el área.

Como hallazgos de este estudio, se destaca que el equipo de enfermería es mayoritariamente responsable de la atención a las AC; incluso en UCI de un hospital con una cultura de seguridad consolidada, se observa la ocurrencia de AC ignoradas y la interrupción de los profesionales durante tareas de enfermería imprescindibles puede comprometer la seguridad del paciente y aumentar la sobrecarga de los profesionales. Tales hallazgos pueden ser útiles para que los enfermeros gestores de UCI optimicen los procesos de trabajo, además de impulsar a los investigadores en el desarrollo de nuevos estudios que evalúen los desenlaces de pacientes expuestos a AC ignoradas, la frecuencia de AC activadas de forma concomitante en UCI y sus efectos sobre la carga de trabajo del equipo de enfermería, así como sobre la seguridad del paciente.

## Conclusión

Se identificaron diversas barreras para la gestión adecuada, incluidas las relacionadas con recursos humanos, factores ambientales y proceso de trabajo. En cuanto a la atención, predominaron las alarmas de monitores multiparámetros, respondidas mayoritariamente por el equipo de enfermería, con una baja proporción de alarmas clínicas ignoradas.

## Data Availability

Los conjuntos de datos relacionados con este artículo estarán disponibles previa solicitud al autor correspondiente.
